# PSMA8‐Containing 20S Proteasome Regulates Spermiogenesis and Male Fertility

**DOI:** 10.1002/advs.202520292

**Published:** 2026-04-13

**Authors:** Huiwen Cao, Qianting Zhang, Wei Xu, Anxuan Fang, Haomang Xu, Cheng Qiu, Lugeng He, Chao Yu

**Affiliations:** ^1^ MOE Key Laboratory of Biosystems Homeostasis & Protection College of Life Sciences Zhejiang University Hangzhou China; ^2^ Zhejiang University‐University of Edinburgh Institute (ZJU‐UoE Institute) Zhejiang University School of Medicine Zhejiang University Haining China; ^3^ The Second Affiliated Hospital Zhejiang University School of Medicine Zhejiang University Hangzhou China; ^4^ Department of Urology the First Affiliated Hospital Zhejiang University School of Medicine Zhejiang University Hangzhou China

**Keywords:** proteasome, PSMA8, s20S, spermiogenesis

## Abstract

Proteasome experiences sophisticated dynamics in types, quantities, and activities during spermiogenesis. However, the physiological importance of proteasomal degradation in mammalian spermiogenesis remains largely unknown. Here, we report that the PSMA8‐containing spermato‐20S proteasome (s20S) is required for spermiogenesis and male fertility in mice. In a mutant mouse model that the C‐terminal 30‐amino‐acid (C30) of PSMA8 is substituted by PSMA7‐C30, the resulting PSMA8^7C30^ protein is unstable, which further disrupts the assembly of s20S and subcellular localization of 19S regulatory particle in testes. *Psma8^7C30^* males could produce round spermatids, but sperm formation is delayed and abnormal, exhibiting a phenotype of oligoasthenoteratozoospermia. s20S is required for the ubiquitination‐dependent proteasomal degradation of a group of proteins in elongating spermatids. These s20S‐mediated degradation events are essential for liquid‐liquid phase separation of FXR1 and thereby translational‐activation of its substrates. Taken together, these findings provide an in vivo evidence of protein homeostasis control in spermiogenesis in mammals.

## Graphical abstract

PSMA8 assembles s20S proteasome that degrades specific substrates in elongating spermatids. Degradation of s20S‐substrates activates translation of FXR1‐target mRNAs, which are essential for mitochondrial sheath formation and sperm morphogenesis. Disruption of s20S in Psma8 mutant mice causes oligoasthenoteratozoospermia and male infertility, demonstrating that proteasomal degradation regulates translational activation during mammalian spermiogenesis.

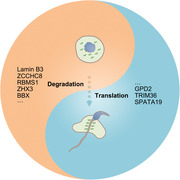

## 1 Introduction

To become spermatozoa capable of fertilizing oocytes, haploid round spermatids, which are produced by spermatocytes through two cycles of cell divisions (meiosis I and meiosis II), undergo a series of highly orchestrated cellular and morphological transformations collectively known as spermiogenesis [1]. These transformations include condensation and compaction of nuclei into the sperm head, biogenesis of acrosome to the anterior and flagellum to the posterior of the sperm head, formation of mitochondrial sheath to the mid‐piece of sperm flagellum, as well as elimination of excessive cytoplasmic components. Therefore, protein homeostasis in spermiogenesis is tightly controlled [2]. On one hand, a group of proteins involved in late spermiogenesis are translated from mRNAs transcribed in early spermiogenesis to regulate specific spermiogenic steps or to participate in the formation of sperm structures [3, 4, 5]. This transcription‐translation decoupling mechanism ensures protein synthesis in elongating spermatids, in which transcription is halted due to nuclear condensation. Notably, while translation is activated by liquid‐liquid phase separation (LLPS) of fragile X‐related protein 1 (FXR1), the mechanism that suppresses translation in round spermatids yet triggers FXR1‐LLPS in elongating spermatids is unknown [3]. On the other hand, another group of proteins are removed through proteasomal degradation, autophagy or into the residual body [6, 7, 8, 9, 10]. However, the mechanism that regulates bulk protein degradation in spermiogenesis, the physiological importance, as well as the interplay between protein degradation and protein translation during spermiogenesis, remain elusive.

Proteasome plays central roles in the ubiquitination‐dependent protein degradation and regulates multiple cellular processes, including cell cycle, cell specification, DNA repair, and senescence [11]. The 20S proteasome core is a 28‐subunit, barrel‐like macromolecule that possesses the caspase‐like, trypsin‐like, and chymotrypsin‐like proteolytic activities [12]. The constitutive 20S (c20S) is made up of four stacked heptameric rings in the arrangement of (α1‐7)(β1‐7)(β1‐7)(α1‐7), forming a catalytic channel to digest protein substrates. Binding of regulatory particles (RPs) alters the proteolytic activities and specificities of 20S. 19S RP (or PA700) is ubiquitously expressed in all cell types and forms the most common 26S proteasome with 20S [13, 14]. Association of PA200 with 20S renders its proteolytic activity to acetylated histones during DNA repair and spermatogenesis [7, 15]. PA28αβ and PA28γ are specifically found in immunocytes and is essential for antigen presentation [16, 17]. However, due to the fundamental roles of c20S in cells and organisms, the in vivo functions of 20S are less investigated.

Testes express a unique isoform of 20S α subunit, PSMA8, which is systematically termed as to α4s [18]. PSMA8 is a paralog of α4 subunit (PSMA7) and could replace PSMA7 in the c20S to assemble a spermato‐20S proteasome (s20S) in male germ cells [19, 20]. PSMA8 protein is detected first in early‐pachytene spermatocytes, increases in late‐pachytene spermatocytes and persists to the end of spermatogenesis [19]. In purified spermatocytes and spermatids, around 80%–90% of 20S proteasomes are s20S; while in spermatogonia and Sertoli cells, 20S proteasomes are almost 100% c20S [20]. c20S regulates the maintenance and differentiation of spermatogonia, whose deficiency leads to arrest of meiosis at the spermatogonial stage [21, 22]. Biochemical assay shows that s20S possesses higher chymotrypsin‐like, trypsin‐like, and caspase‐like protease activities than c20S, both in the presence and absence of PA200 [20]. Furthermore, s20S and c20S exhibit divergent binding affinity to different RPs in testes. The amount of 19S is found to be 14.5‐fold higher in the anti‐PSMA8 immunoprecipitant than in the anti‐PSMA7 immunoprecipitant. As a consequence, higher proteasome activity is evident in lysates derived from spermatocytes and spermatids [20]. s20S is essential for male fertility, as revealed by several *Psma8* knockout mouse models [19, 23, 24]. s20S targets histones and many prophase I proteins (such as RAD51, RPA1, SYCP3, etc.) for degradation in spermatocytes, so that spermatogenesis in *Psma8* mutant males are arrested at the metaphase I stage, which limits the investigation of s20S function in spermatids [19, 23, 24]. Here, we generated two *Psma8* mutant mouse models overcoming the metaphase I‐arrest and found that s20S‐mediated protein degradation is essential for FXR1‐LLPS and translation of FXR1‐targets, which further regulates sperm elongation and male fertility.

## 2 Results

### 2.1 PSMA8 is Required for Degradation of Ubiquitinated Proteins in Spermatids

To explore the functions of proteasomal degradation in spermiogenesis, we analyzed the patterns of protein ubiquitination and proteasome composition in mouse testes (Figure S1). The lysine 48‐linkage specific ubiquitin (Ub‐K48) accumulates at mid‐pachytene and peaks in step‐10 elongating spermatids (ES10), indicating high ubiquitination and protein degradation during spermatid elongation (Figure S1A). Consistently, the level of 20S, which is represented by an antibody against α subunits (α‐sub), is constantly high in spermatocytes and spermatids (Figure S1B). While PSMA7 has relatively higher expression level in cells close to the lamina of seminiferous epithelia (spermatogonia and leptotene/zygotene spermatocytes), PSMA8 is predominantly expressed in pachytene/diplotene spermatocytes and spermatids (Figure S1B,C), confirming that s20S is the predominant 20S species in these cells.

**FIGURE 1:**
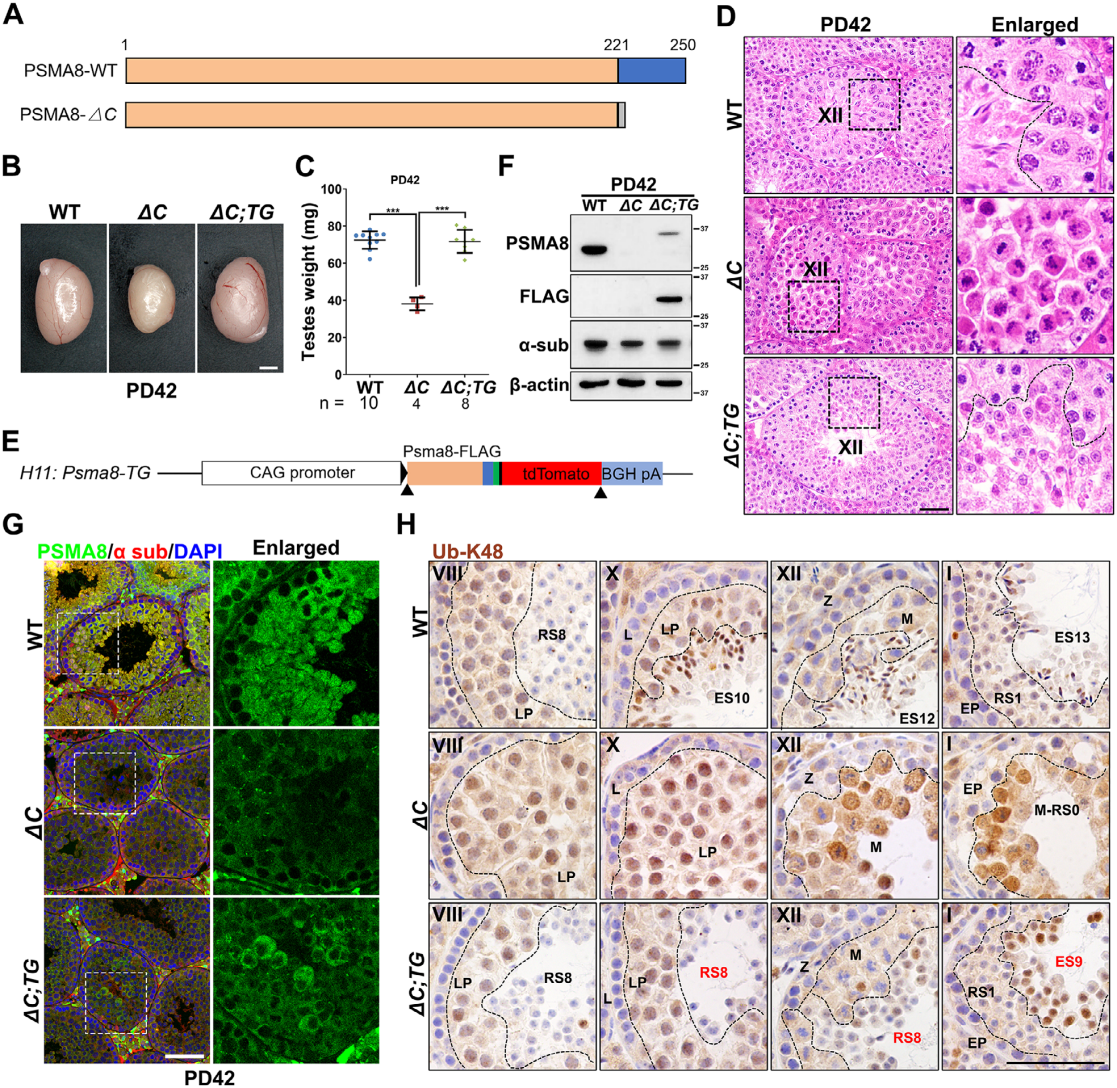
*ΔC;TG* males overcome the metaphase I‐arrest phenotype in *Psma8 ΔC* males. (A) Schematic diagram depicting the PSMA8^WT^ and PSMA8^ΔC^ proteins. (B,C) A representative image of WT, *ΔC* and *ΔC;TG* testes at PD42 (B) and the weight of these testes are shown in (C). Scale bar, 2 mm. Error bars indicate S.D. n indicates the numbers of testes analyzed. ^***^, *p* < 0.001 (two‐tailed Student's *t* test). (D) Hematoxylin & eosin (HE) staining of testes sections derived from WT, *ΔC* and *ΔC;TG* males at PD42. Scale bar, 50 µm. (E) Schematic diagram depicting the strategy to generate *Psma8‐TG* knock‐in allele at the *H11* locus. The translation start codon and stop codon (black triangles) are indicated. (F) Western blotting data of antibodies against indicated proteins in testes samples derived from WT, *ΔC* and *ΔC;TG* males. (G) Co‐staining of PSMA8 (green) and α‐subunits (α‐sub, red) in PD42 testes. Scale bar, 50 µm. (H) IHC staining of Ub‐K48 in testes sections derived from WT, *ΔC* and *ΔC;TG* males at PD42. Roman numerals indicate the stages of seminiferous tubules. L, leptonema; Z, Zygonema; EP, early‐pachynema; LP, late‐pachynema; M, metaphase; RS, round spermatids; ES, elongating spermatids; SP, Spermatozoa. Scale bar, 50 µm.

By comparison of the amino acid sequences of PSMA7 and PSMA8 among various species, we identified a C‐terminal 30 amino acids (C30) motif as the least conserved region between PSMA7 and PSMA8 (Figure S2). To investigate the functions of s20S in spermiogenesis, we first generated a *Psma8^ΔC^* allele, in which a 247‐base pair (bp)‐region spanning 3’‐intron 6 and 5’‐exon 7 (which encodes PSMA8‐C30) is deleted (Figure 1A; Figure S3A,B). Homozygous *Psma8^ΔC/ΔC^* males (termed as *ΔC*) are infertile and phenocopy a previously reported *Psma8‐KO* males [19] in testes size (Figure 1B,C) and metaphase I‐arrest (Figure 1D) at postnatal day 42 (PD42).

We then generated a *Psma8* transgenic model (*Psma8‐TG* or *TG*) in which a cassette containing *Psma8‐FLAG*, *P2A*, and *tdTomato* under the control of a *CAG* promoter is inserted into the *H11* locus (Figure 1E). *Psma8‐TG* expresses PSMA8‐FLAG in testes, however, at a relatively low level, when compared to endogenous PSMA8 in WT testes (Figure 1F,G). Strikingly, the *ΔC;TG* males (*Psma8^ΔC^* males carrying the *Psma8^TG^* allele) have testes appearing normally in size and weight (Figure 1B,C). Histology analyses show that elongating spermatids at step‐10 are observed in *ΔC;TG* testes (Figure 1D), suggesting that a trace PSMA8 level (rendered by PSMA8‐TG) is sufficient to overcome the metaphase I‐arrest phenotype observed in *Psma8^Δ^*. Moreover, while spermatocytes at late‐pachytene and metaphase I in *Psma8^ΔC^* males accumulate high level protein polyubiquitination (Ub‐K48), this Ub‐K48 level is decreased in *ΔC;TG* germ cells at similar stages (Figure 1H). Elongating spermatids in *ΔC;TG* testes accumulates high levels of Ub‐K48 (Figure 1H) and mature spermatozoa are not observed in *ΔC;TG* epididymes (Figure S3C), suggesting that normal s20S activity is required for spermiogenesis.

**FIGURE 2:**
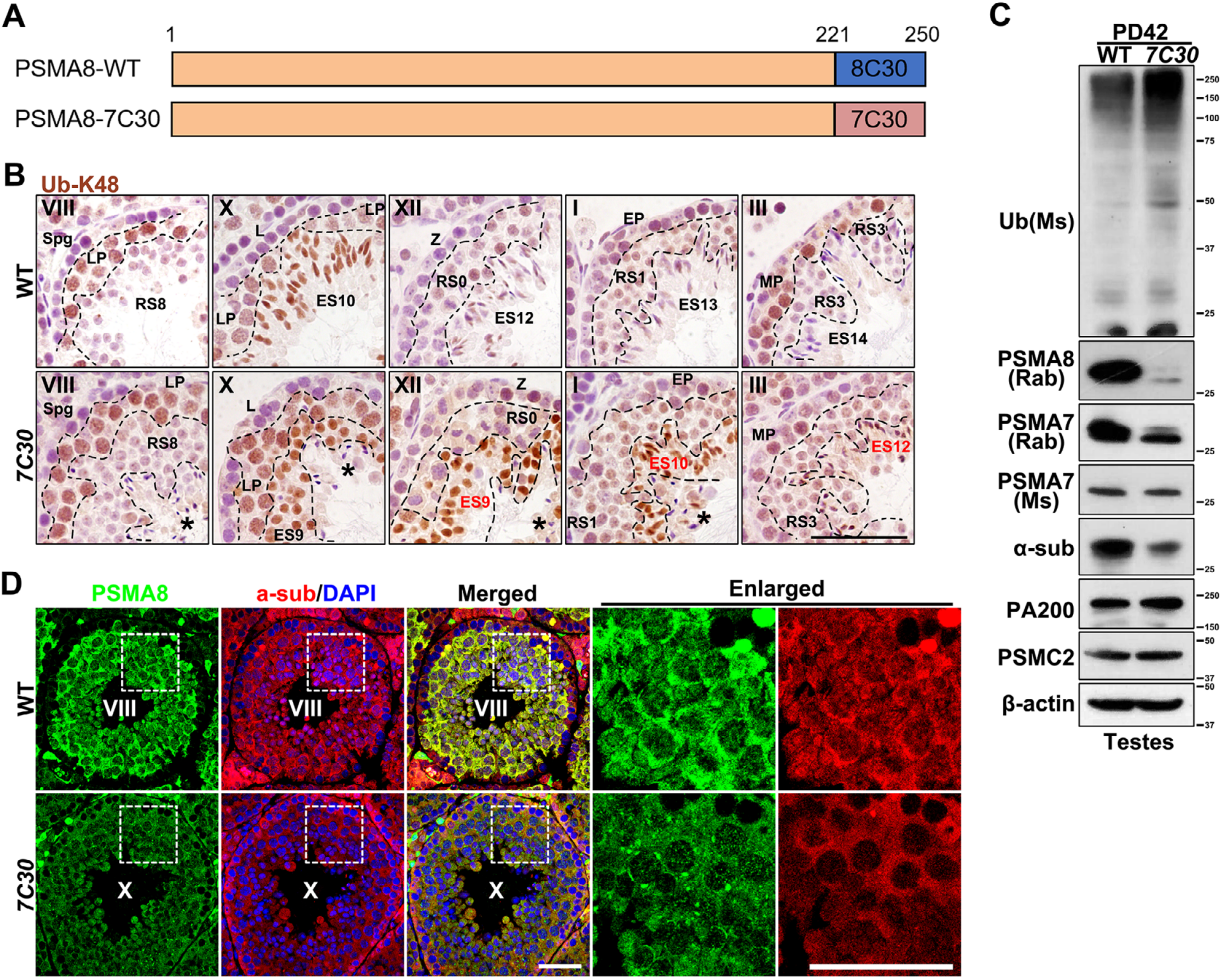
*7C30* is a hypomorphic mutation of *Psma8* that phenocopies *ΔC;TG*. (A) Schematic diagram depicting the PSMA8^WT^ and PSMA8^7C30^ proteins. (B) Immunohistochemical (IHC) staining of Ub‐K48 in testes sections derived from WT and *7C30* males. Spg, spermatogonia; MP, mid‐pachynema; Asterisks indicate abnormally retained spermatids of the previous spermatogenic cycle. Scale bar, 50 µm. (C) Western blotting data showing the levels of indicated proteins in WT and *7C30* testes at PD42. Rab, Rabbit; Ms, Mouse. (D) Immunofluorescent (IF) staining of PSMA8 (green) and proteasomal α subunits (α‐sub, red) in WT and *7C30* testes sections at PD42. The enlarged images show regions bordered with dashed lines. Scale bars, 50 µm.

Because *Psma8^ΔC^* resembles *Psma8^KO^* in metaphase I‐arrest, we then substituted PSMA8‐C30 with PSMA7‐C30 and constructed a *Psma8‐7C30* knockin allele in which PSMA8‐C30 is replaced by a cassette containing sequences encoding PSMA7‐C30, P2A, and CRE‐GFP (Figure 2A; Figure S4A,B). The resulting *Psma8^7C30^* allele expresses a PSMA8^7C30^ mutant protein and a CRE‐GFP recombinant protein. *Psma8^7C30/+^* and *Psma8^7C30/7C30^* (termed as *7C30* thereafter) mice exhibit no overt phenotypes with respect to viability, growth, and external morphology (Figure S4C,D). Interestingly, *7C30* testes do have a number of spermatids, however, the level of Ub‐K48 is dramatically increased in elongating spermatids in stage X/XII/I seminiferous tubules (Figure 2B,C). Therefore, *Psma8^ΔC;TG^* and *Psma8^7C30^* mouse models enable the investigation of s20S functions in spermiogenesis and suggest that PSMA8, or s20S, is required for the ubiquitination‐dependent protein degradation in spermiogenesis.

### 2.2 s20S is Essential for Spermiogenesis and Male Fertility in Mice

*Psma8^7C30/+^* males are crossed with *H11^mGmT/mGmT^* females, and the resulting *Psma8^7C30/+^;H11^mGmT/+^* males (termed as *mGmT;7C30*) are subjected to assess the activity of CRE recombinase. As shown by tdTomato fluorescence, CRE recombinase activity is exerted in the germ cells of *mGmT;7C30* testes at PD42 (Figure S4E), indicating the effectiveness of *Psma8* promoter‐driven CRE expression by the knock‐in allele. Quantitative RT‐PCR results reveal that the level of *Psma8^7C30^* transcripts in *7C30* testes is significantly reduced compared to *Psma8^WT^* transcripts in WT testes (Figure S4 F,I). Consistent with the reduced mRNA level, the level of PSMA8^7C30^ mutant protein, detected by an antibody against both PSMA8^WT^ and PSMA8^7C30^, is dramatically decreased in *7C30* testes (Figure 2C,D; Figure S4G,H), which may result from decreased transcriptional efficiency and/or reduced mRNA stability. As a result, the amount of proteasome α subunits is decreased in *7C30* testes (Figure 2C,D; Figure S4H). Since more than 80% proteasomes in spermatocytes and spermatids are composed of s20S [20], we could conclude that the decreased level of PSMA8^7C30^ leads to defective s20S assembly in testes.

s20S disassembly is also observed in previously reported *Psma8^KO^* mouse model, in which spermatogenesis is arrested at metaphase I with aberrant accumulation of RAD51 and RPA1 [19]. The *Psma8^7C30^* model is different from the *Psma8^KO^* model in: 1) the meiotic progression into metaphase I is not obviously affected in *Psma8^7C30^*, as evidenced by the comparable percentages of seminiferous tubules containing phospho‐histone H3 (pHH3) in WT and *7C30* testes (Figure S5A,B); 2) the protein levels of RAD51, RPA1 and SYCP3 are not increased in *7C30* testes at PD16 and PD21 (Figure S5C); and 3) the existence of spermatids in *7C30* testes (Figure 2B,D). By contrary, similar to the *Psma8^KO^* model, meiotic recombination in *7C30* males is compromisingly affected (Figure S5D–G). Noting that in *7C30* testes, PSMA8^7C30^ is detected at a trace level (Figure 2C,D) and similarly in *ΔC;TG* testes, PSMA8‐TG is expressed at a relatively low level (Figure 1F,G), we postulate that remnant level of s20S activity supports completion of meiosis I and II to produce haploid spermatids.

**FIGURE 3:**
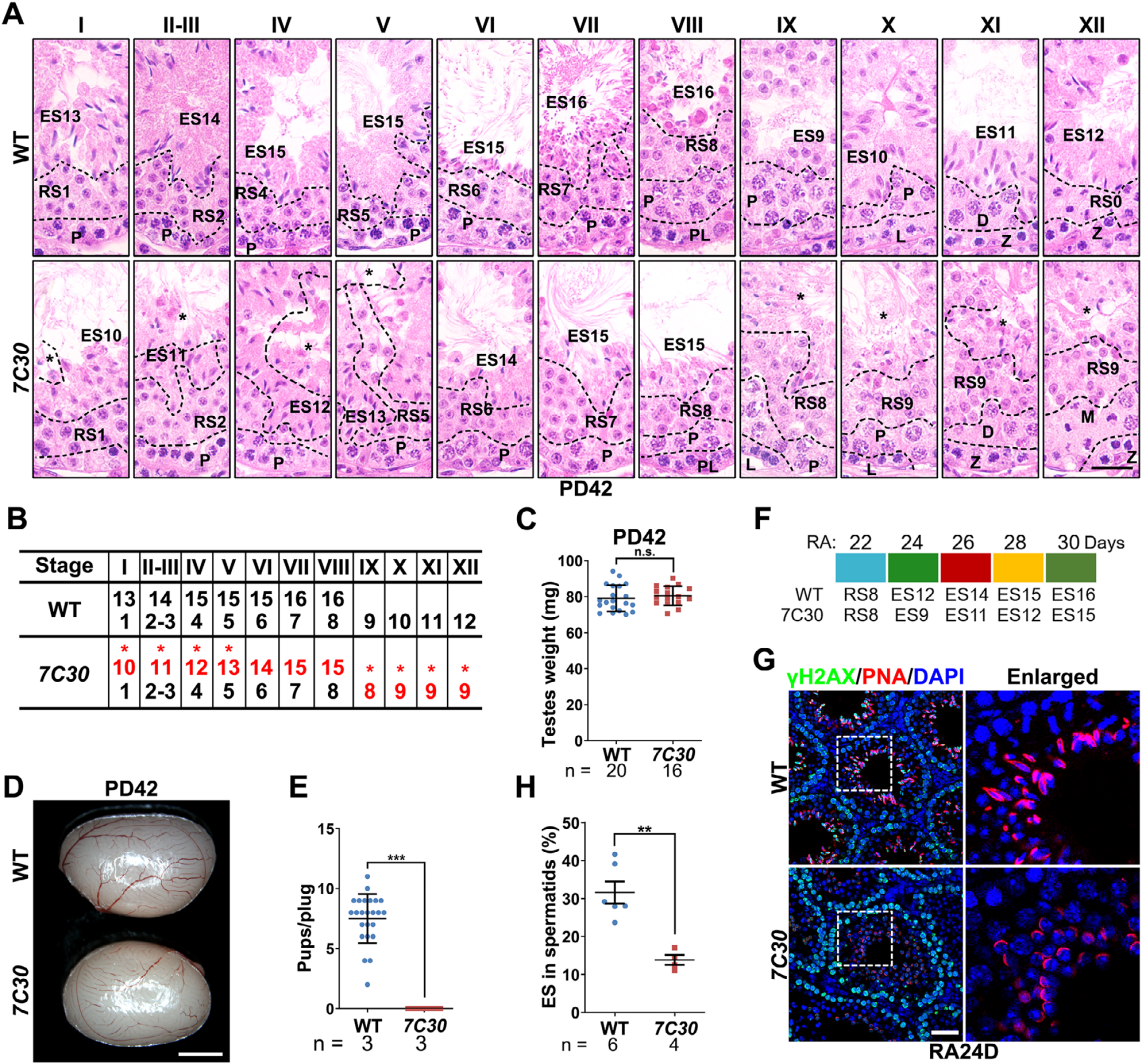
*7C30* male mice are infertile with delayed spermiogenesis. (A) Hematoxylin & Eosin (H&E) staining analyses of testes sections derived from WT and *7C30* males at PD42. PL, pre‐leptonema; P, pachynema. Scale bar, 20 µm. (B) Summary of spermatid types in stage I‐XII seminiferous tubules in PD42 WT, *7C30* and *7C30;TG* testes. Asterisks indicate abnormally retained spermatids of the previous spermatogenic cycle. (C) Weight of testes derived from WT and *7C30* males at PD42. Error bars indicate S.D. n indicates the numbers of male mice analyzed. n.s., not significant. ^***^, *p* < 0.001 (two‐tailed Student's *t* test). (D) A representative image of WT and *7C30* testes at PD42. Scale bar, 2 mm. (E) Fertility test of WT and *7C30* males. Data was shown as pups per plug. Error bars indicate S.D. n indicates the numbers of testes analyzed. ^***^, *p* < 0.001 (two‐tailed Student's *t* test). (F) Schematic diagram showing the progression of spermiogenesis in synchronized WT and *7C30* males after RA induction. (G) Co‐staining of γH2AX (green) and PNA (red) showing the morphology of spermatids in WT and *7C30* testes at 24 days post RA administration (RA24D). Scale bar, 50 µm. (H) Percentage of elongated spermatids (ES) in haploid cells in WT and *7C30* testes. Error bars indicate S.D. n indicates the numbers of mice analyzed. ^**^, *p* < 0.01 (two‐tailed Student's *t* test).

However, histological analyses of the stages of seminiferous tubules in WT and *7C30* testes demonstrate delayed spermiogenesis in *7C30* spermatids (Figure 3A,B). The difference becomes distinguishable first in stage IX seminiferous tubules where WT spermatids start to elongate (Figure 3A,B). Widely‐distributed morphologically abnormal spermatids undergoing apoptosis are detected in *7C30* testes (Figure S6A,B). Testes derived from *7C30* males appear in normal size and have similar weight to WT controls (Figure 3C,D), which is different from the 60% testis‐weight decrease in *Psma8^ΔC^* and *Psma8^KO^* males at PD42. Fertility test results show that, while *7C30* females exhibit normal fertility (Figure S4C), *7C30* males are totally infertile (Figure 3E). We then used a WIN 18 446‐retinoic acid (RA)‐combined spermatogenesis synchronization protocol to analyze the elongation process of *7C30* spermatids [25, 26]. WT and *7C30* testes are comparable at 20 days post RA administration (RA20D) and RA22D (Figure S4J), indicating that meiosis and spermatid development in *7C30* testes are normal and synchronized with WT before RA24D. At RA22D, both WT and *7C30* spermatids are at round spermatid step‐8, or RS8. However, while WT spermatids progress to elongating spermatid step‐12 (ES12) at RA24D, *7C30* spermatids only progress to step‐9 with their nuclear remaining relatively round (Figure 3F,G). Fluorescence‐activated cell sorting (FACS) analyses show that the ratio of elongating spermatids in 1N spermatids is decreased in *7C30* testes at RA24D (Figure 3H; Figure S6C,D). Massive apoptosis is not detected in *7C30* testes at RA24D and RA26D; however, elongating spermatids undergo apoptosis in *7C30* testes at RA28D and RA30D (Figure S6E,F). These results suggest that s20S is required for late spermiogenesis and male infertility in mice.

### 2.3 Defective Mitochondrial Sheath Formation in *7C30* Spermatozoa

**FIGURE 4:**
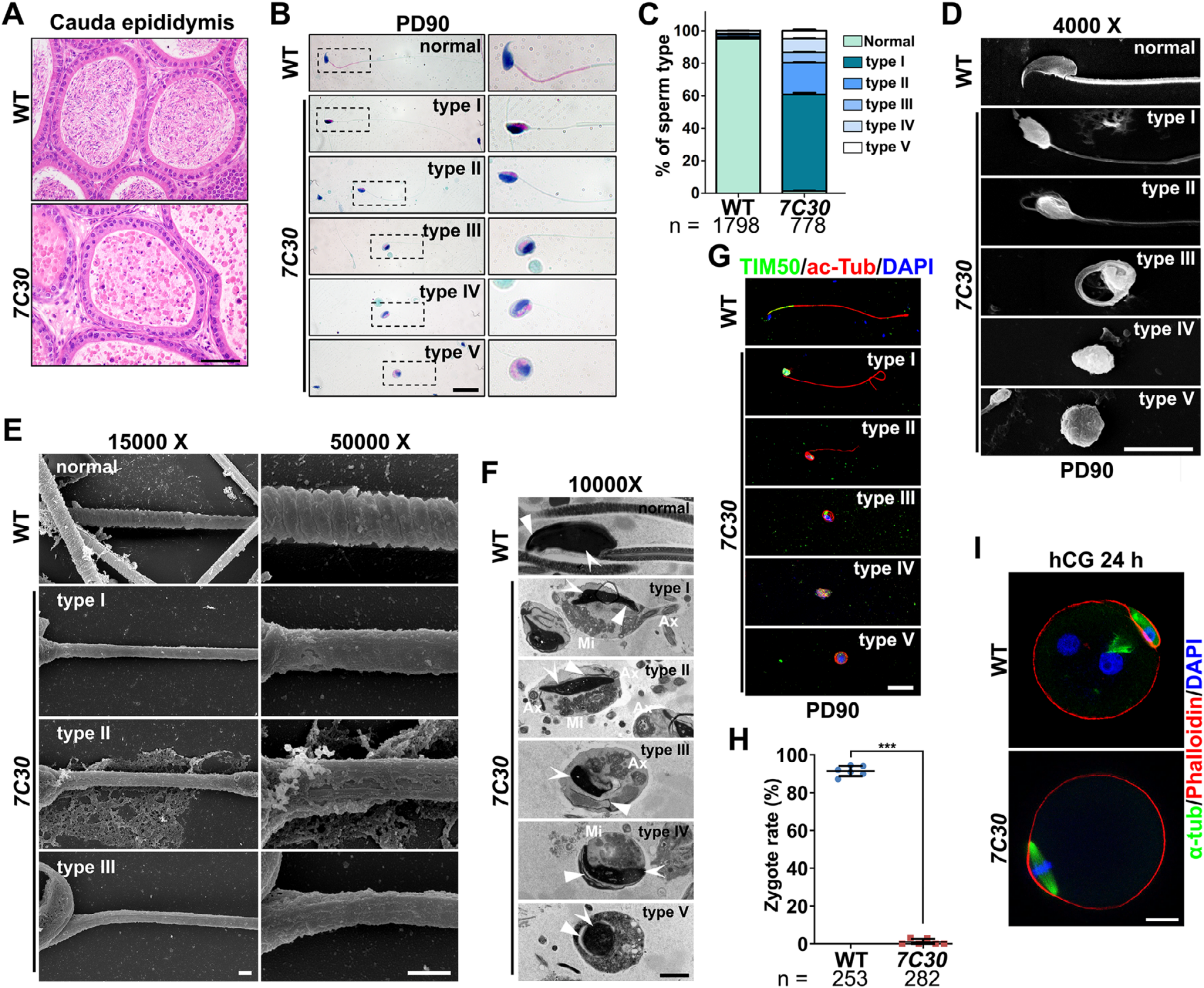
Defective mitochondrial sheath formation in *7C30* spermatozoa. (A) H&E staining of cauda epididymis derived from WT and *7C30* males at PD42. Scale bar, 50 µm. (B) Papanicolaou staining of spermatozoa derived from the epididymis of WT and *7C30* males. Scale bar, 20 µm. (C) Percentages of spermatozoa types in WT and *7C30* males. Error bars indicate S.E.M. n indicates the numbers of oocytes analyzed. (D) Scanning electric microscopy (SEM) images showing the morphology of spermatozoa derived from WT and *7C30* epididymis. Scale bars, 10 µm. (E) SEM images showing mitochondrial missing in the mid‐piece of *7C30* spermatozoa. Scale bars, 500 nm. (F) Transmission electric microscopy (TEM) images showing abnormal mitochondrial aggregations in sperm head. The nuclear and acrosomes are indicated by open‐end and blunt‐end arrowheads, respectively. Mi, Mitochondria; Ax, Axoneme. Scale bar, 2 µm. (G) IF staining of TIM50 (a mitochondrial inner membrane protein, green) and acetylated α‐tubulin (ac‐tub, red) in WT and *7C30* spermatozoa. Scale bar, 20 µm. (H,I) In vitro fertilization (IVF) experiments showing that *7C30* spermatozoa are uncapable of fertilizing MII oocytes. The spindles (green) and F‐actin (red) are immune‐stained by FITC‐α‐tubulin and rhodamine‐phalloidin, respectively (I) and the percentages of oocytes fertilized (zygote rate) are shown in (H). Scale bar, 20 µm. Error bars indicate S.D. n indicates the numbers of oocytes analyzed. ^***^, *p* < 0.001 (two‐tailed Student's *t* test).

As the result of spermiogenesis defects, the cauda epididymes of *7C30* males are full of morphologically‐abnormal cells with condensed or uncondensed nuclei (Figure 4A). Spermatozoa derived from the epididymes of WT and *7C30* males were analyzed by computer‐assisted sperm analysis (CASA; Figure S7A). Compared to the WT controls that have an amount of 11.37 ± 0.57 million spermatozoa, the number of spermatozoa in *7C30* males is significantly decreased to 0.81 ± 0.20 million (p = 2.33 × 10^−6^), which is only 7.1% of WT controls (Figure S7A). Moreover, the motility of *7C30* spermatozoa is also largely damaged, as characterized by the percentage of motile sperm (86.48 ± 1.40 in WT *vs*. 7.65 ± 1.55 in *7C30*), the percentage of progressively motile sperm (68.75 ± 1.84 in WT *vs*. 0 in *7C30*), the curvilinear velocity (67.83 ± 2.51 µm/s in WT *vs*. 6.90 ± 2.82 µm/s in *7C30*), the straight‐line velocity (18.80 ± 1.20 µm/s in WT *vs*. 2.18 ± 0.91 µm/s in *7C30*) and the average path velocity (34.08 ± 1.51 µm/s in WT *vs*. 3.80 ± 1.91 µm/s in *7C30*; Figure S7A).

The morphology of spermatozoa was further analyzed by papanicolaou staining and immunostaining of acetylated‐α‐tubulin (ac‐Tub, a marker of flagellum) with PNA [27]. WT spermatozoa have a normal morphology, as manifested by an acrosome‐coated sickle‐shaped sperm head and a long sperm flagellum with the mitochondrial sheath alongside the mid‐piece of the flagellum (Figure 4B,C; Figure S7B). However, *7C30* spermatozoa exhibit morphological abnormities including spindle‐shaped sperm nuclei with remnant of cytoplasm, short/bent/coiled/absent sperm flagella, as well as frequent absence of mitochondrial sheath (Figure 4B,C; Figure S7B). These abnormities are also observed in the scanning electron microcopy (SEM) images of *7C30* spermatozoa (Figure 4D).

Specifically, the mitochondrial sheath is commonly absent in *7C30* spermatozoa (Figure 4D,E). Instead, mitochondria are ectopically accumulated in the head of spermatozoa, as examined by transmission electron microcopy (TEM) and fluorescent staining of mitochondrial proteins (Figure 4F,G; Figure S7C,D). Furthermore, the axonemes of *7C30* spermatozoa are frequently found structurally incomplete (Figure S7E–G). Therefore, we conclude that *7C30* males are reduced in the quantality and motility with morphological abnormities, which is collectively known as a phenotype of OAT. As a consequence, spermatozoa derived from *7C30* cauda epididymes are not able to fertilize WT oocytes at the metaphase II stage (Figure 4H,I; Figure S7H).

### 2.4 Deregulated Proteasome Assembly and Protein Homeostasis in *7C30* Mutants

**FIGURE 5:**
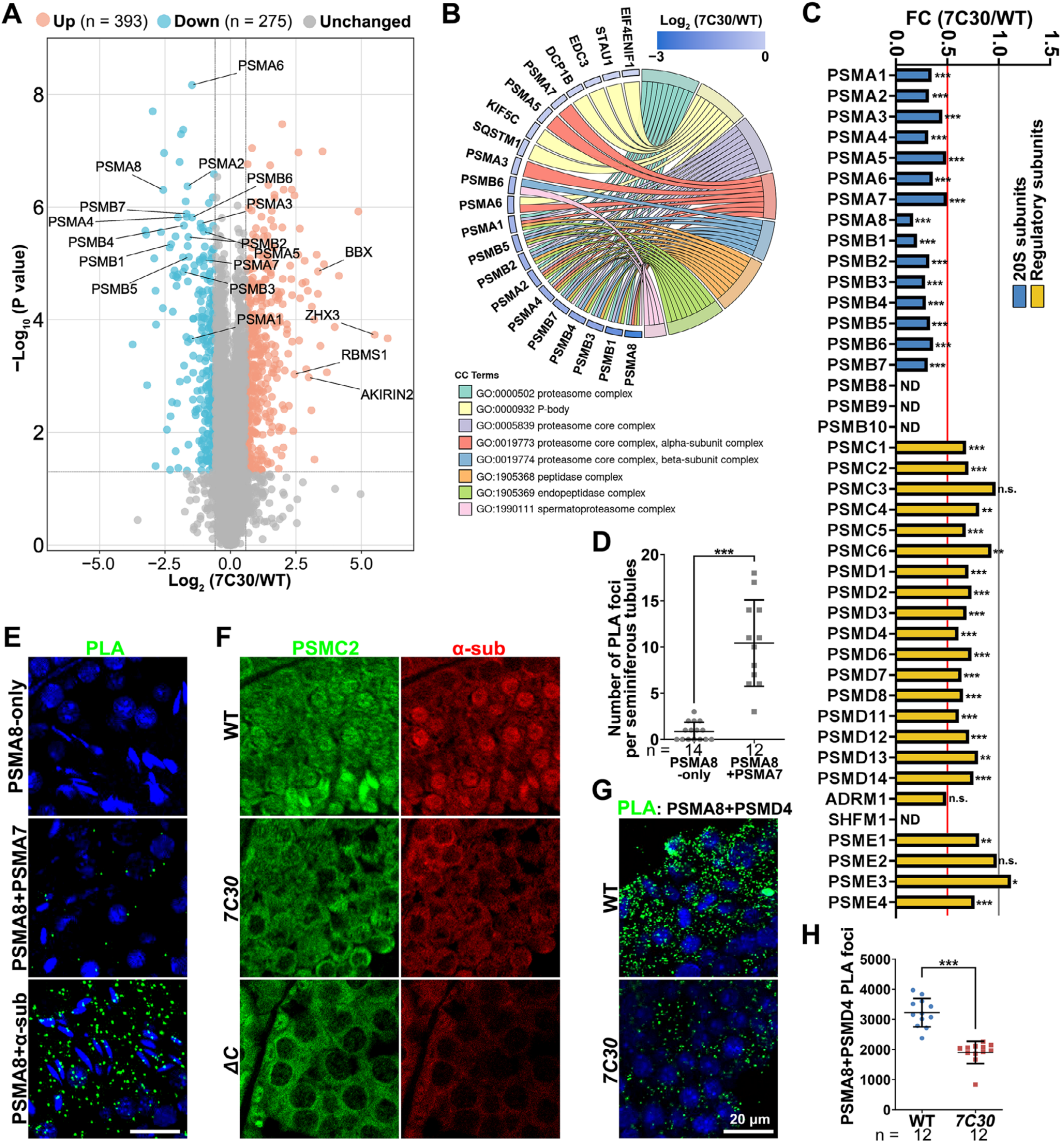
Proteomic analysis of elongating spermatids in WT and *7C30* testes. (A) Proteomic changes in purified *7C30* spermatids at RA24D, as plotted by Log_2_ (*7C30*/WT) and –Log_10_ (*p* value). (B) Gene Ontology enrichment analyses of down‐regulated proteins in *7C30* spermatids. (C) The variation in protein levels of proteasomal subunits in *7C30* spermatids. Data was retrieved from the MS results. ND, not detected. n.s., not significant. ^**^, *p* < 0.01; ^***^, *p* < 0.001 (two‐tailed Student's *t* test). (D) Quantification of the numbers of PLA foci in each seminiferous tubules with indicated antibody combinations. Error bars indicate S.D. n indicates the numbers of seminiferous tubules analyzed. ^***^, *p* < 0.001 (two‐tailed Student's *t* test). (E) Proximity ligation assay (PLA) with indicated antibody combinations in WT testes at PD42. Scale bar, 20 µm. (F) Staining of PSMC2 (green) and α subunits (α‐sub, red) in *Psma8^7C30^* and *Psma8^ΔC^* testes. Scale bar, 20 µm. (G) PLA with a combination of PSMA8 (rabbit) and PSMD4 (mouse) antibodies in WT and *7C30* testes at RA24D. Scale bar, 20 µm. (H) Quantification of PSMA8/PSMD4 PLA foci in each seminiferous tubules in WT and *7C30* testes at RA24D. Error bars indicate S.D. n indicates the numbers of seminiferous tubules analyzed. ^***^, *p* < 0.001 (two‐tailed Student's *t* test).

To understand the mechanisms of why s20S disruption impairs spermiogenesis, we sorted elongating spermatids from synchronized WT and *7C30* testes at RA24D and subjected them for proteomic analyses (Figure 5A; Figure S8). A total number of 5090 proteins are identified in quantitative mass spectrometry (MS) results, among which 393 proteins are up‐regulated (Fold change > 1.5, *p* < 0.05) and 275 proteins are down‐regulated in *7C30* spermatids (Fold change > 1.5, *p* < 0.05; Figure 5A; Table S1). For fold change (FC) > 2, the numbers of proteins up‐ and down‐regulated are 200 and 109, respectively (Figure S8A,B). GO assay shows that the down‐regulated proteins (FC > 1.5) are enriched in the terms of proteasome core complexes, which is consistent with the observation of s20S disruption in *7C30* testes (Figure S8B,C). To be more detailed, all components of the 20S subunits (PSMA1‐8 and PSMB1‐7) are down‐regulated in *7C30* spermatids (Figure 5B,C; Figure S9).

It is not surprising that PSMA8 is down‐regulated because PSMA8^7C30^ protein is not stable (Figure 2D). Down‐regulation of other 20S components suggests defective s20S assembly in *7C30* spermatids (Figure 2C,D; Figure 5B,C). Interestingly, the protein level of PSMA7 is also decreased to 50% in *7C30* spermatids (Figure 5C; Figure S9), indicating the formation of a type of chimeric 20S proteasome consisting of both PSMA7 and PSMA8 in spermatids, which is also destabilized due to instability of PSMA8^7C30^. To test this possibility, we performed proximity ligation assay (PLA) with a combination of PSMA8 and PSMA7 antibodies (PSMA8+PSMA7) in WT testes sections (Figure 5D,E). PLA foci of the positive control (PSMA8+α‐sub) is extensively detected in seminiferous tubules while PLA signal of the negative control (PSMA8‐only) is barely observed. An average number of 10.42 ± 1.35 PLA foci are detected in the group of PSMA8+PSMA7, confirming the presence of chimeric 20S proteasome in testes (Figure 5D,E).

Furthermore, many components of RPs are also down‐regulated, although at a relatively lower degree, in *7C30* spermatids (Figure 5C; Figure S9). Compared to an average 2.95‐fold decrease of 20S subunits, RPs are decreased by 1.32‐fold on average. As a result, in Western blotting results, the protein levels of PA200 and PSMC2 (Rpt1) in *7C30* testes are comparable to WT controls (Figure 2C). However, the subcellular localization of PSMC2 is disrupted in *7C30* spermatocytes and spermatids (Figure 5F). In WT spermatids, PSMC2 and α subunits are accumulated dominantly in the nuclei; however, in *7C30* spermatids at the same stage, the level of 20S α subunits is decreased and PSMC2 localizes exclusively to the cytoplasm (Figure 5F). The subcellular localization of another 19S subunit, PSMD4 (Rpn10), is similarly affected in *7C30* testes (Figure S10A). Decreased PLA foci of PSMA8+PSMD4 also suggests impaired s20S‐19S proteasome assembly in *7C30* testes (Figure 5G,H). The aberrant subcellular localization of 19S subunits is also observed in spermatocytes of *Psma8^ΔC^* testes (Figure 5F). On the contrary, the subcellular localization of PA200 is not affected in *7C30* testes (Figures S9 and S10B).

**FIGURE 6:**
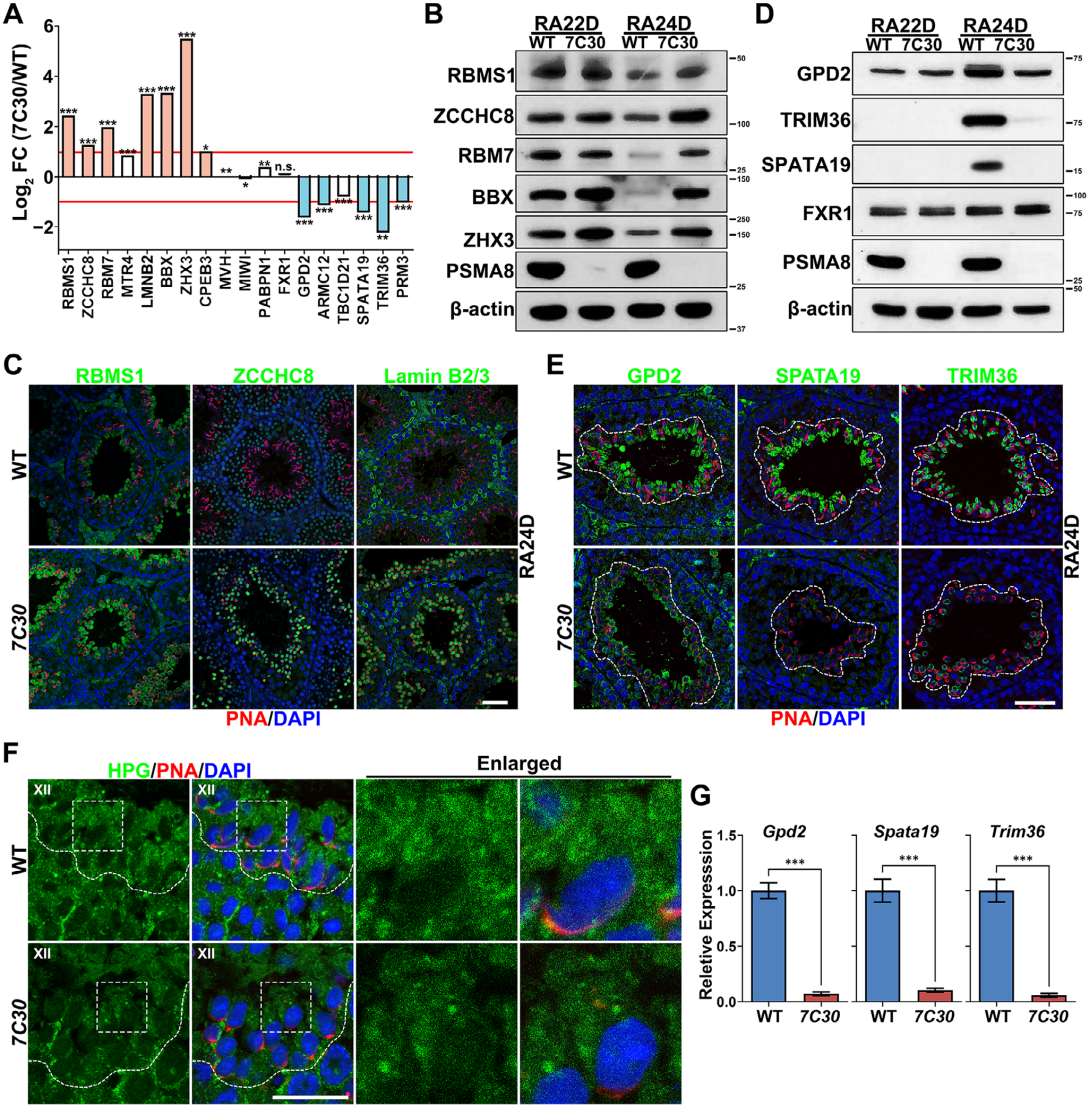
Deregulated protein homeostasis in *7C30* mutants. (A) The variation of indicated protein levels in *7C30* spermatids. Data was retrieved from the MS results. n.s., not significant. ^*^, *p* < 0.05; ^**^, *p* < 0.01 and ^***^, *p* < 0.001 (two‐tailed Student's *t* test). (B,C) Western blotting (B) and IF staining (C) verifying the protein level of some up‐regulated proteins (RBMS1, ZCCHC8 and Lamin B2/3) in WT and *7C30* spermatids at RA22D and RA24D. Scale bar, 50 µm. (D,E) Western blotting (D) and IF staining (E) verifying the changes in protein level of down‐regulated proteins in WT and *7C30* spermatids at RA22D and RA24D. Scale bar, 50 µm. (F) The levels of translation was labeled with HPG in elongating spermatids within testes sections of WT and *7C30* male mice at PD30. Scale bar, 25 µm. (G) qRT‐PCR analyses of the levels of ribosome‐bound *Gpd2*, *Spata19* and *Trim36* mRNAs in polysome fraction. The mRNA levels in WT is set as “1”. ^***^, *p* < 0.001 (two‐tailed Student's *t* test).

On the other hand, we are more interested in the up‐regulated proteins, because they are potential s20S‐substrates that should be degraded in normal spermiogenesis, however, stabilized in *7C30* spermatids (Figure 5A; Figure S8). These proteins are not enriched into a few specific categories by GO analysis (Figure S8B,D). Instead, they are related to diverse biological processes, such as sperm structures (acrosome, nuclear lamina, sperm connecting piece), E3 ubiquitin ligases (SCF ubiquitin ligase), RNA processing (TRAMP complex, RNA polymerase II), etc. (Figure 6A; Figure S8B,D). We then verified the protein levels of some notable candidates, including RBMS1 [28], ZCCHC8 [29], Lamin B2/3 [30], BBX, and ZHX3 (Figure 6B,C; Figure S10C,D). Their protein levels are usually comparable in WT and *7C30* testes at RA22D, and decreased in WT testes at RA24D (Figure 6B,C). However, their protein levels remain high in *7C30* testes at RA24D, suggesting that they are s20S‐substrates. We have also identified a group of candidates for c20S‐substrates, including MVH, PABPN1, and MIWI, which are normally degraded in elongating spermatids in both WT and *7C30* testes (Figure 6A; Figure S11).

### 2.5 Impaired Translation of FXR1‐Targets in *7C30* Elongating Spermatids

Among the down‐regulated proteins (FC > 2) in *7C30* spermatids, 54.1% (59 out of 109 proteins; Table S2) have been previously identified as FXR1 targets by eCLIP‐seq [3]. Furthermore, 30 out of the 59 FXR1‐targets are decreased in protein levels in FXR1‐null elongating spermatids. Many of these proteins are involved in sperm elongation during spermiogenesis, such as GPD2 [31], ARMC12 [32, 33], CST8 [34], SPATA19 [35], and TRIM36 [3, 36, 37].

GPD2 is a well‐established substrate of FXR1 in sperm elongation [3, 31], and is decreased at an FC of 3.08 in *7C30* spermatids at RA24D (Figure 6, A,D,E). ARMC12 and its interactor TBC1D21 are both required for mitochondrial sheath formation in the mid‐piece of spermatozoa, and their loss‐of‐function mutations cause male infertility in both human and mice [32, 33, 38]. SPATA19 is also critical for mitochondrial sheath formation in elongating spermatids [35]. In *7C30* spermatids, ARMC12, TBC1D21, and SPATA19 are decreased at 2.19, 1.73, and 2.68‐fold, respectively (Figure 6A), Similar defects in mitochondrial sheath formation is observed in *7C30* spermatids (Figure 4; Figure S7). TRIM36, or haprin, which is involved in the acrosome reaction [37], is 4.69‐fold down‐regulated in *7C30* spermatids (Figure 6A). The decreased protein levels of GPD2, SPATA19, and TRIM36 in elongating *7C30* spermatids are also confirmed by immunofluorescent staining and Western blotting (Figure 6D,E). To visualize the impact of the *7C30* mutation on translation in elongating spermatids, we performed HPG labeling to measure translation efficiency. Robust HPG signals were detected in the cytoplasm of WT ES12 spermatids, whereas *7C30* spermatids displayed only sparse punctate signals (Figure 6F). Moreover, we performed sucrose gradient fractionation and qRT‐PCR to detect the ribosome‐bound mRNAs in the polysome fraction of WT and *7C30* testes. As shown in Figure 6G, the mRNA levels of *Gpd2*, *Spata19*, and *Trim36* were dramatically decreased in *7C30* testes. These results indicate that the *7C30* mutation impairs overall translation activity in elongating spermatids, consistent with the reduced expression of FXR1‐target proteins.

### 2.6 Proteasomal Degradation is a Prerequisite for LLPS of FXR1

**FIGURE 7:**
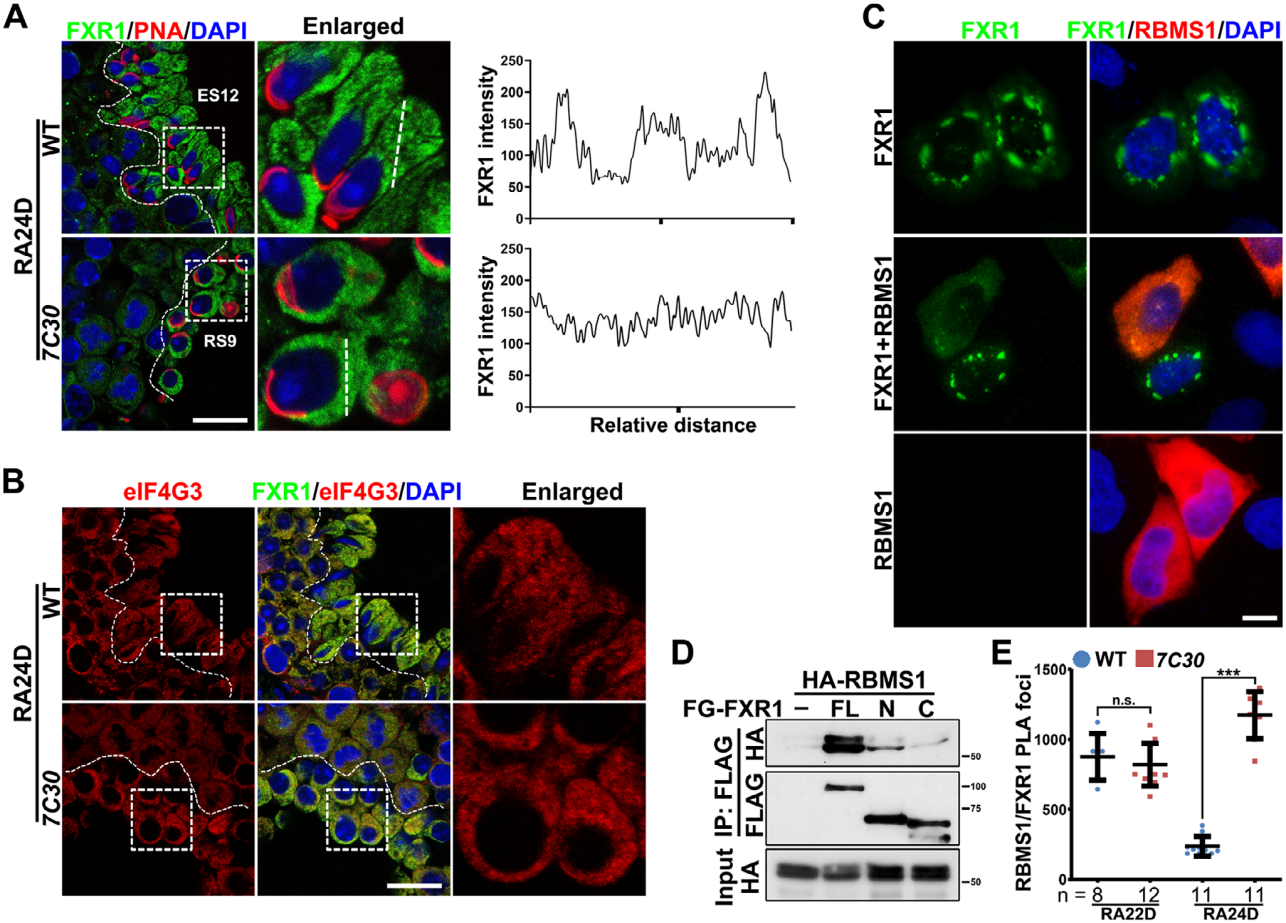
s20S‐mediated proteasomal degradation is required for LLPS of FXR1. (A) IF staining of FXR1 (green) in testes sections derived from WT and *7C30* males at RA24D. The FXR1 intensities in single elongating spermatids were line‐plotted on the right. Scale bar, 20 µm. (B) Co‐staining of FXR1 (green) and eIF4G3 (red) showing their defective LLPS in *7C30* spermatids at RA24D. Scale bar, 20 µm. (C) RBMS1 inhibits the LLPS of FXR1 in HeLa cells. Scale bar, 10 µm. (D) Immunoprecipitation experiments showing the interaction of RBMS1 with FXR1. The size of protein markers is indicated. FG, FLAG‐EGFP; FL, full‐length of FXR1a; N, N‐terminal of FXR1a; C, C‐terminal of FXR1d. (E) Quantification of RBMS1/FXR1 PLA foci in each seminiferous tubules. Error bars indicate S.D. n indicates the numbers of seminiferous tubules analyzed. ^***^, *p* < 0.001 and n.s., *p* > 0.05 (two‐tailed Student's *t* test).

LLPS of FXR1 is required for the translational activation of many spermiogenesis‐specific proteins during spermiogenesis [3]. Due to the down‐regulation of FXR1‐targets in elongating spermatids in *7C30* testes, we sought out to investigate the status of FXR1‐LLPS in WT and *7C30* testes. LLPS of FXR1 occurs in WT elongating spermatids; however, FXR1 distributes more evenly in the cytoplasm in *7C30* spermatids at RA24D, suggesting that LLPS of FXR1 may be impaired in *7C30* elongating spermatids (Figure 7A; Figure S12A,B). This pattern is also observed in stage IX‐XII seminiferous tubules in PD42 testes (Figure S12C). The *7C30* sperm cytoplasm exhibits reduced FXR1 punctation signals. Similarly, co‐staining of FXR1 and eIFG3 showed that the phase‐separated eIFG3 is reduced in *7C30* spermatids at RA24D (Figure 7B).

Among the s20S substrates, RBMS1 is an RNA binding protein and is involved in the translational control of its target mRNAs [39]. Moreover, RBMS1 has been identified as an FXR1‐interacting protein [40]. In elongating WT spermatids at RA24D, the protein level of RBMS1 decreases and FXR1 undergoes LLPS (Figure S12D). However, in elongating *7C30* spermatids at the same stage, the protein level of RBMS1 remains high and FXR1 is less phase‐separated (Figure S12D). Interestingly, when transfected into HeLa cells, expression of RBMS1 leads to an even distribution of FXR1 (Figure 7C), suggesting a role of RBMS1 in inhibiting the LLPS of FXR1. PLA of RBMS1 and Ub‐FK2 on WT and *7C30* testes at RA24D shows that RBMS1 can be ubiquitinated, and the increased foci in *7C30* testes indicates accumulated ubiquitinated RBMS1 (Figure S12F,G), supporting that RBMS1 is degraded through ubiquitination and proteasome pathway. Co‐immunoprecipitation experiments confirm that RBMS1 interacts with FXR1 (Figure 7D). This RBMS1‐FXR1 interaction is also observed in PLA results in WT and *7C30* testes (Figure 7E; Figure S12E). RBMS1 and FXR1 are closely co‐localized in both WT and *7C30* spermatids at RA22D before elongation (Figure 7E; Figure S12E). The number of RBMS1+FXR1 PLA foci is dramatically decreased in WT spermatids at RA24D, however, it remains high in *7C30* spermatids at the same stage (Figure 7E; Figure S12E). Therefore, we propose that RBMS1 may inhibit LLPS of FXR1 in round spermatids, whereas proteasomal degradation of RBMS1 by s20S may be a prerequisite for the LLPS of FXR1 in elongating spermatids.

## 3 Discussion

Our results demonstrated a pivotal role of proteasomal degradation on translational activation of spermiogenic‐specific proteins in spermiogenesis, the post‐meiotic phase of spermatogenesis. s20S directs proteasomal degradation of a group of proteins, known as s20S‐substrates, in elongating spermatids, and stabilization of which collectively disables a spermiogenic‐specific protein translational activation machinery that triggered by LLPS of FXR1 (Figure S13A). As a consequence, many FXR1 substrates that are commonly required for the processes of spermiogenesis are inadequately translated in *Psma8^7C30^* spermatids. Therefore, s20S controls protein homeostasis in spermatids through: 1) assembly of 20S proteasome as well as recruitment of RPs to 20S; 2) proteasomal degradation of s20S substrates; and 3) translation of FXR1‐activated spermiogenic proteins.

Transformation of the mitochondrial network into the mitochondrial sheath is a hallmark of spermiogenic progression [41, 42]. However, due to the fundamental roles of mitochondrial dynamic‐related proteins, conditional ablation of *Fis1* or *Mfn1/2* in spermatocytes leads to mitochondrial dysfunction and spermatogenic arrest at meiotic prophase I [41, 43]. Therefore, control of mitochondrial sheath formation in spermiogenesis is less understood. Specifically, mitochondrial sheath formation is completely impaired in *7C30* spermatozoa, resulting in spermatozoa lacking of mitochondrial sheath in the mid‐piece of flagella (type I) or spermatozoa with ectopically accumulated mitochondrial in the head (type I, II, III, and IV). Even though mitochondria‐surrounded axoneme structures are observed in a number of *7C30* spermatozoa, these structures are loose and consist of a heap of morphological irregular mitochondria (Figure 4F; Figure S7C). Similar phenotypes have been observed in knockout or mutant of *Armc12*, *Tbc1d21*, and *Spata19*. Interestingly, defective mitochondrial sheath formation could be explained by insufficient translation of ARMC12, TBC1D21, and SPATA19 in elongating *7C30* spermatids (Figure 6D,E). Besides, other 28 FXR1‐targets are also insufficiently translated in *7C30* spermatids (Table S2). Among them, the deletion of GPD2, CST8 or TRIM36 leads to male infertility [31, 34, 36, 44].

PSMA8 is a known substitute of α4 in the 20S proteasome and is expressed at a high level from pachytene/diplotene spermatocytes to the end of spermatogenesis [18]. However, due to the indispensable roles of s20S in meiotic prophase I, PSMA8‐null male germ cells are arrested at metaphase I with a DNA content of 4N. By generating a *Psma8^7C30^* mutant model and a *Psma8^ΔC;TG^* rescue model, which could produce haploid spermatids with a DNA content of 1N, we are possible to investigate the physiological functions of s20S as well as protein homeostasis control in spermiogenesis in this study (Figure S13B). Although PSMA8^7C30^ is detected at a trace level (Figure 2C), the remnant PSMA8^7C30^‐20S proteasomes are sufficient to degrade the previously identified s20S substrates in meiotic prophase I (Figure S5C), thereby enabling the completion of meiosis I and meiosis II, as well as the investigation of s20S functions in spermiogenesis (Figure S13B). However, due to the mutational nature of PSMA8^7C30^, the possibility of gain‐of‐function should be taken into consideration. During the period of this study, heterozygous *Psma8^7C30/+^* males expressing both PSMA8^WT^ and PSMA8^7C30^ are fertile with no defects in spermiogenesis. To rule out this possibility, we also investigated whether weak PSMA8‐TG expression could rescue the phenotype of metaphase I arrest in *Psma8^ΔC^*. As shown in Figure 1, *ΔC;TG* males could produce elongating spermatids, suggesting that the trace activity of s20S in *7C30* and *ΔC;TG* is sufficient for the completion of meiosis I and meiosis II.

Although both PSMA8 and PA200 (encoded by *Psme4*) are required for spermiogenesis, the phenotypes caused by their deficiencies are different [7, 45]. The spermiogenic steps in *Psme4* knockout testes are compromisingly affected and the main cause of infertility is the failure in removing core histones in elongating spermatids [7, 45]. However, the primary phenotype of *Psma8^7C30^* males is OAT originated from defective morphological transformation in elongating spermatids, suggesting that PA200 might not be the major RP in spermatids. This discovery provides physiological evidence to a recent study that uncovered 19S as the major RP of s20S in spermatocytes and spermatids [20]. 19S occupies 60% of total proteasomes in spermatids, while PA200 is only found in 5% proteasomes. Components of 19S are enriched at a level of 14.5‐fold more in anti‐s20S immunoprecipitant than in anti‐c20S immunoprecipitant. Strikingly, in *7C30* spermatocytes and spermatids, the subcellular localization of 19S (as shown by PSMC2) rather than PA200 is disrupted. Interestingly, the complexity of proteasome composition in testes is far more beyond alternative RPs that associate with c20S or s20S. We have also identified a small proportion of chimeric 20S consisting of both PSMA7 and PSMA8 subunits by PLA (Figure 5D,E). In theory, if PSMA7 and PSMA8 form c20S and s20S separately, the level of PSMA7 should not be affected in *7C30* testes. Therefore, down‐regulation of PSMA7 also indicates the presence of chimeric 20S proteasomes that are made up of one α ring with PSMA7 and the other α ring with PSMA8. These chimeric 20S is also disrupted due to the instability of PSMA8^7C30^.

Except for s20S substrates, we found a group of proteins such as MVH [46, 47], PABPN1 [48], and MIWI [49, 50], are normally degraded in elongating spermatids in *7C30* testes (Figure S11), indicating the involvement of c20S in spermiogenesis. The differences between c20S and s20S could be expressed, at least, by following two aspects. First, s20S possesses higher protease activities than c20S [20]. Second, s20S and c20S exhibit diverse binding affinities to proteasome interactors and potential substrates. Taken together, our findings suggest a model that s20S‐regulated protein homeostasis control, through protein degradation and protein translation, is critical for spermatid elongation in spermiogenesis in mammals.

## 4 Experimental Section

### 4.1 Mice

The *Psma8^7C30^* knock‐in mice (carrying the *7C30* mutant allele of *Psma8*) and the *H11^Psma8‐LSL^* mice (with a *CAG‐LSL‐Psma8‐FLAG‐P2A‐tdTomato* transgenic cassette at *H11* locus) were separately generated at the GemPharmatech LLC relying on the CRISPR‐Cas9 technique. The homogenous *Psma8^7C30/7C30^* mice (referred to as *7C30*) were obtained by crossing *Psma8^7C30/+^* males and females. In the germline of *Psma8^7C30/+^; H11^Psma8‐LSL/+^* mice, obtained by crossing *Psma8^7C30/+^* with *H11^Psma8‐LSL/PSMA8‐LSL^* mice, the STOP codon within the LSL sequence was deleted, leading to a genetic‐modified allele carrying a constitutively active cassette expressing exogenous PSMA8‐FLAG (referred to as *H11^Psma8‐TG^*). Therefore, *Psma8^7C30/7C30^;H11^Psma8‐TG/+^* ^or^ *^Psma8‐TG/ Psma8‐TG^* mice (both referred to as *7C30*;TG) were generated through the breeding of *Psma8^7C30/+^;H11^Psma8‐LSL/+^* males and females. In other cases, *Psma8^7C30/+^* mice were crossed to mGmT knock‐in mice (GemPharmatech LLC) to validate the CRE recombinase activity. The *Psma8* knockout mouse stain (*Psma8^ΔC^*) was homemade by the i‐GONAD methods [51]. The strategies for designing the gene‐modified alleles in these mouse strains were described in relevant figures. The genotyping primers are listed in Table S3. The animal care and experimental procedures were conducted under the supervision of Laboratory Animal Welfare and Ethics Committee of Zhejiang University (ZJU20220056). The mice were housed in SPF laboratory animal facilities with the feeding conditions set as 20°C–22°C, 12 h light/dark cycle and 50%–70% humidity.

### 4.2 Fertility Test

Each male mice from all experimental groups (WT, *7C30*, *TG*, and *7C30;TG*) were crossed to two wild‐type (WT) females in a cage from the age of 6‐week‐old. The cages were maintained for more than 3 months to monitor the presence of vaginal plugs and pregnancy. The number of pups born and sex ratios for all litters were carefully recorded to calculate the fertility.

### 4.3 Sperm Assay

The cauda epididymides were dissected and incubated in the human tubal fluid (HTF) medium at 37°C for the dispersion and capacitation of spermatozoa. The parameters of spermatozoa motility were assessed with a computer‐assisted sperm analysis (CASA) system at 60 min after incubation. For morphological analyses, spermatozoa suspensions were spread onto adhesive microscopy slides. After air‐dry, the samples were fixed in 4% PFA for 30 min and then subjected to papanicolaou staining or immunofluorescent staining.

### 4.4 In Vitro Fertilization (IVF)

For MII oocytes collection, PD21‐23 females were subjected to a superovulation procedure, that is intraperitoneally injection with a dose of PMSG (pregnant mare serum gonadotrophin, 5 IU/10 g) for 44–48 h and then a dose of hCG (human chronic gonadotrophin, 5 IU/10 g). Cumulus‐oocyte complexes (COCs) were collected from the oviducts at 16 h post hCG administration. On the day of IVF, spermatozoa obtained from cauda epididymides and vas deferens were incubated in HTF medium, as described above. After 60 min of incubation, COCs were collected and cultured in HTF drops covered with mineral oil, and then spermatozoa were added to trigger fertilization. 6 h after IVF, oocytes/zygotes were extensively washed and transferred to KSOM medium to allow further embryogenesis.

### 4.5 Synchronization of Spermatogenesis

Synchronization of spermatogenesis was performed with newborn males using WIN 18 446 and retinoic acid (RA), as previously reported [25]. WIN 18 446 was suspended in 1% gum tragacanth at a concentration of 50 mg/ml. RA was prepared in DMSO at a concentration of 20 mg/ml. Newborn males were pipette‐fed WIN 18 446 at a dose of 100 µg/g body weight once a day from PD2 to PD8. After 7 doses of WIN 18 446 (i.e., PD9), RA were intraperitoneally injected at a dose of 22 µg/g. Male mice were sacrificed at different time points after RA administration for the analyses of spermatogenesis.

### 4.6 Isolation of Male Germ Cells

FACS (fluorescence‐activated cell sorting) method was used to isolate specific populations of male germ cells in testes, based on Hoechst‐33342 staining plotted with Hoechst red and Hoechst blue on a BD Influx Cell Sorter (BD Biosciences) [52]. Briefly, seminiferous tubules were incubated first in PBS containing collagenase type I at 32°C for 10 min, and then in 0.25% trypsin‐EDTA with DNase I at 32°C for 5 min. FBS were added to terminate digestion and cells were filtrated through a 70 µm BD Falcon cell strainer. Isolated total male germ cells were resuspended in DMEM medium and stained with Hoechst‐33342 (at a concentration of 5 µg/10^6^ cells) at 32°C for 30 min. Propidium iodide (PI, 1 µg/10^6^ cells) were added immediately prior to sorting to exclude dead cells.

### 4.7 Histological Analyses

For H&E staining, testes and epididymides were fixed in Bouin's solution overnight, and then rinsed in 50% and 70% ethanol to remove the yellow color. For immunofluorescent (IF) staining and immunohistochemical (IHC) staining, tissues were fixed in PBS buffered 3.7% formaldehyde. The samples were further dehydrated with an ethanol gradient, xylene, and paraffin and finally embedded in paraffin for preparation of 5‐µm thick sections. Paraffin‐embedded sections were deparaffinized with xylene, rehydrated with an alcohol gradient and rinsed with water. For H&E staining, sections were stained with hematoxylin and eosin respectively (30–60 s each). For IHC staining, dehydrated sections were subjected to peroxidase blocking in 3% hydrogen peroxide for 10 min, thereafter boiled in 10 mm Sodium Citrate Buffer (pH 6.0) at 95°C for 15 min for antigen unmasking and then cooled to room temperature on bench. The slides were sequentially incubated with blocking buffer (10% goat serum in PBS containing 0.1% Tween‐20) for 30 min, primary antibody dilutions in blocking buffer for 60 min, biotinylated secondary antibody dilutions for 30 min, streptavidin‐HRP complexes in the Vectastain ABC Kit for 30 min and finally stained with DAB Peroxidase Substrate Kit (Vector Laboratories). The primary antibodies involved are listed in Table S4.

### 4.8 Immunofluorescent (IF) Staining

Paraffin‐embedded sections were deparaffinized, rehydrated, and antigen unmasked as in for IHC staining. Afterward, the slides were successively incubated in Blocking buffer (1% BSA in PBS with 0.1% Tween‐20) for 30 min, in diluted primary antibody for 60 min and in diluted Fluorophores (FITC and CY3)‐conjugated secondary antibody (Jackson ImmunoResearch) containing 5 µg/ml DAPI for 60 min. The stained slides were mounted with 80% glycerol and imaged with a confocal laser scanning microscope (Olympus, FV3000). The antibodies involved are listed in Table S4.

### 4.9 Nuclear Surface Spreading

Nuclear surface spreadings were performed as described previously [53]. Briefly, seminiferous tubules derived from male testes were immersed in Hypotonic Extraction Buffer (30 mm Tris‐HCl, 50 mm sucrose, 17 mm trisodium citrate dehydrate, 5 mm EDTA, and 0.5 mm DTT, pH8.2) for 30 min and thoroughly smashed with sharp forceps in 100 mm Sucrose Solution (pH 8.2) to disperse testicular cells. An aliquot of 20 µl cell suspension was added on the surface of a slide containing Fixative Buffer (1% paraformaldehyde and 0.15% Triton X‐100, pH 9.2). The slides were kept in a humidify box for 2 h to become air‐dried and subjected to immunofluorescent staining.

### 4.10 TUNEL Assay

TUNEL assays were conducted following the protocol of TUNEL BrightGreen Apoptosis Detection Kit (Vazyme, #A112‐01). Briefly, the paraffin‐embedded sections were deparaffinized and rehydrated, followed by Proteinase K digestion, FITC‐12‐dUTP labeling, contra‐staining with DAPI and mounting. The imaging process is in the same way as IF assay. Finally, the FITC‐positive cells in seminiferous tubules were quantified for statistical analysis.

### 4.11 Semi‐Quantitative and Quantitative RT‐PCR

Total RNA was purified from tissues dissected from male mice at indicated ages with RNeasy Mini Kit (Qiagen, #74104), and cDNA was synthesized with PrimeScript II first Strand cDNA Synthesis Kit (TaKaRa, #6210A). Semi‐quantitative PCR amplification was carried out with 2 × Taq DNA Polymerase Mix following standard procedures for 22–30 cycles. Quantitative RT‐PCR (qRT‐PCR) was performed using the same reverse‐transcribed cDNA with SYBR Green Premix Pro Taq HS qPCR Kit (Accurate Biology, #AG11701) according to the manufacturer's instructions. The PCR primers designed for amplifying *Psma8* and *Gapdh* cDNA fragments are listed in Table S4.

### 4.12 cDNA Cloning

cDNAs encoding *Psma7* or *Psma8* were amplified from mouse testes cDNA library by high‐fidelity PCR polymerase and inserted into a pCAG‐GFP expressing vectors via restriction enzyme digestion cloning protocols. *Rbms1* cDNA were cloned into pRK5‐HA vector. *Fxr1* (isoform a and d) and its truncations were cloned into pRK5‐FLAG‐GFP vector. The successful constructs were further verified by DNA sequencing.

### 4.13 Western Blotting

Protein extracts were collected from testes and denatured in SDS loading buffer (25 mm Tris‐HCl, 2% SDS, 10% glycerol, 5% β‐mercaptoethonol, 0.01% bromophenol blue, pH 6.8), assisted with thoroughly sonication. Protein samples were separated by SDS‐polyacrylamide gel electrophoresis (SDS‐PAGE), and transferred to PVDF membrane with Trans‐Blot SD Semi‐Dry Transfer Cell (Bio‐Rad). After sequential incubation of the membrane with diluted primary antibody and HRP‐conjugated secondary antibody (Jackson ImmunoResearch), chemiluminescent signals were developed with SuperSignal West Pico PLUS (Thermo Fisher, #34577) and images were acquired with Odyssey FC Western Blot Imaging System (LI‐COR Biosciences). The antibodies applied are listed in Table S4.

### 4.14 Cell Lines and Transfection

HEK‐293T and HeLa cells were cultured in DMEM high glucose medium (Sigma–Aldrich, #D6546) supplemented with 10% fetal bovine serum (FBS) under a condition of 37°C, 5% CO_2_. Cells were transfected with plasmids carrying GFP‐tagged proteins via Lipofectamine 2000 (Invitrogen, #11668019), and harvested after 24 h. Whole cell lysates were extracted with nuclear lysis buffer (25 mm Tris‐HCl, 300 mm NaCl, 1% Triton X‐100, 1 mm DTT, 1 mm EDTA, pH 7.5, with protease inhibitors freshly supplemented), afterward boiled in SDS sample buffer at 95°C for 10 min and subjected for Western blotting analyses.

### 4.15 Immunoprecipitation

Transient‐transfected HEK‐293T cells were extracted with IP Binding Buffer (25 mm Tris‐HCl, 150 mm NaCl, 1% NP‐40, 1 mm DTT, 1 mm EDTA, pH 7.5, supplemented with protease inhibitors). An aliquot of one‐tenth whole cell lysate was boiled in SDS loading buffer as Input. The remnant cell lysate was incubated with ANTI‐FLAG M2 Affinity Gel (Sigma, #A2220) on a rotating shaker at 4°C for 4 h. Afterward, the beads bound with immuno‐precipitated proteins were washed thoroughly in IP Washing Buffer (25 mm Tris‐HCl, 350 mm NaCl, 1% Triton X‐100, 1 mm DTT, 1 mm EDTA, pH 7.5), with the supernatant removed by pipet and boiled in SDS loading buffer as IP sample. The Input and IP samples were further examined by Western blotting.

### 4.16 Scanning Electron Microscopy (SEM)

Cauda spermatozoa were initially processed with two‐step fixation procedure: first fixed in 2.5% glutaraldehyde (diluted in 0.1 m phosphate buffer, pH 7.0) for over 4 h, and washed thoroughly in PBS for 3 times/15 min; second fixed in 1% OsO4 (diluted in PBS) for 1–2 h and washed again. Then the samples were dehydrated through a series of gradient ethanol solutions. After dehydration in Hitachi Model HCP‐2 critical point dryer, the samples were coated with gold‐palladium in Hitachi Model E‐1010 ion sputter for 4–5 min, and finally scanned under a Hitachi Model SU‐8010 SEM.

### 4.17 Transmission Electron Microscopy (TEM)

The complete process of TEM was conducted at the Analysis Center of Agrobiology and Environmental Sciences, Zhejiang University. Sample processing procedures for TEM are similar to that of SEM. After fixation, the samples were serially dehydrated by an ethanol gradient (30%, 50%, 70%, 80%) and an acetone gradient (90%, 95%, 100%, 100%) for 15–20 min for each step. After that, the samples were sequentially infiltrated in a mixture of Spurr resin and acetone (V/V = 1/1) for 1 h, a mixture of Spurr resin and acetone (V/V = 1/3) for 3 h and absolute Spurr resin for overnight, followed by embedding in Spurr resin at 70°C for overnight. Ultrathin sectioning was performed with a LEICA EM UC7 ultratome and stained with alkaline lead citrate solution and uranyl acetate (saturated solution in 50% ethanol) for 5–10 min, respectively. Finally, specimens were observed and imaged with a Hitachi Model H‐7650 TEM or a JEOL 2100 plus TEM (JEOL, Tokyo, Japan).

### 4.18 Mass Spectrometry (MS) Analyses

MS experiments and analyses were performed at the PTM BIO, Hangzhou. Briefly, tissue samples were grinded with liquid nitrogen, lysed in a lysis buffer (8 m urea, 1% protease inhibitor cocktail), followed by sonication. After centrifugation, the supernatant was collected and the protein concentration was determined with BCA kit. Trypsin was added at 1:50 trypsin‐to‐protein mass ratio for the first digestion overnight and 1:100 trypsin‐to‐protein mass ratio for a second 4‐hour‐digestion. Finally, the peptides were desalted by C18 SPE column and loaded to a timsTOF Pro (Bruker Daltonics) mass spectrometry. The electrospray voltage applied was 1.60 kV. Precursors and fragments were analyzed at the TOF detector, with a MS/MS scan range from 100 to 1700 m/z. GO annotation is to annotate and analyze the identified proteins with eggnog‐mapper software (v2.0). Extracting the GO ID from the results of each protein note, and then classified the protein according to Cellular Component, Molecular Function and Biological Process.

### 4.19 HPG Treatment and Detection

HPG (L‐homopropargylglycine) treatment was performed based on established protocols [54]. HPG (Beyotime, #ST2057) was dissolved in PBS at 10 mg/mL and stored at −80°C until use. Mice received a single intraperitoneal injection of HPG at 0.1 mg/g body weight. Testes were harvested 24 h post‐injection and processed for analysis as described above. For HPG signal detection, click reaction (Beyotime, #P1202S) was performed according to the manufacturer's instructions.

### 4.20 Sucrose Gradient Analysis and Polysome Fractionation

Sucrose gradient analysis of testicular extracts was performed with slight modifications based on a previously described protocol [3]. Mouse testicular tissues were homogenized in extraction buffer containing 100 mm NaCl, 50 mm Tris‐HCl (pH 7.5), 5 mm MgCl_2_, 1% Triton X‐100, and 100 µg/ml cycloheximide, followed by centrifugation at 13 000 *g* for 2 min at 4°C to remove nuclear pellets. The resulting supernatants were layered onto 10%–60% linear sucrose gradients and centrifuged at 38 000 rpm using a P40ST rotor for 2 h and 15 min at 4°C. Fractions were collected from top to bottom, and each fraction was subjected to Western blot analysis. The RNA from the polysome fractions were extracted as previously described [55], followed by RT‐qPCR for absolute quantification of the indicated transcripts using the standard curve method. To draw the standard curve, DNA templates for each target gene were amplified from mouse testis cDNA using ApexHF HS DNA Polymerase FS (Accurate Biology, AG12202). The copy number of each template was calculated using the formula: copy number (copies/µL) = Avogadro's constant (6.02 × 10^2^ ^3^ copies/mol) × concentration (g/µL) / PCR product molecular weight (g/mol). The templates were then serially diluted at 10‐fold intervals to generate an 8‐point standard curve ranging from approximately 10^3^–10^10^ copies/µL.

### 4.21 Statistical Analysis

For each experiment, at least three independent experiments were carried out with similar results. Otherwise indicated, for experiments with mice or mice samples, at least three mice were analyzed. The statistical data were processed and exhibited as means ± S.D. or means ± S.E.M. The significance of differences between two independent groups were analyzed by two‐tailed unbiased Student's *t* test. The level of statistical significance is expressed as *p* < 0.05 (^*^), *p* < 0.01 (^**^), *p* < 0.001 (^***^) or *p* > 0.05 (n.s.).

## Author Contributions

C.Y. designed the project. C.Y., Q.Z. and H.C. performed most of the experiments and analyzed the data with assistance of A.F., C.Q. and L.H. W.X. did the FACS‐based germ cell sorting. H.X. acquired the TEM and SEM images. Q.Z. and C.Y. wrote the manuscript with help from all authors. All authors read and approved this manuscript.

## Conflicts of Interest

The authors declare no conflicts of interest.

## Supporting information




**Supporting File 1**: advs75270‐sup‐0001‐SuppMat.docx.

**Supporting File 2**: advs75270‐sup‐0002‐Table S1.xlsx.

**Supporting File 3**: advs75270‐sup‐0003‐Table S2.xlsx

## Data Availability

The data that support the findings of this study are available in the supplementary material of this article.
