# Supplementary material for: PSMA8‐Containing 20S Proteasome Regulates Spermiogenesis and Male Fertility
Source: Adv Sci (Weinh). 2026 Apr 13;13(39):e20292. doi: 10.1002/advs.202520292 (PMC13334872; doi:10.1002/advs.202520292)
Supplement: Supplementary file 1 — Supporting File 1: advs75270‐sup‐0001‐SuppMat.docx. [file ADVS-13-e20292-s003.docx]

**The PDF file includes:**

Figs. S1 to S13

Tables S3 and S4

**Other Supplementary Material for this manuscript includes the following:**

**Table S1.** Proteins identified by quantitative mass spectrometry (MS) in WT and *7C30* spermatids at RA24D.

**Table S2.** Lists of proteins that are up-regulated or down-regulated in *7C30* spermatids, down-regulated proteins that are FXR1-targets by eCLIP, down-regulated proteins that are also decreased in FXR1 cKO spermatids, and identified FXR1-targes.

**Supplementary Figures and legends**

**
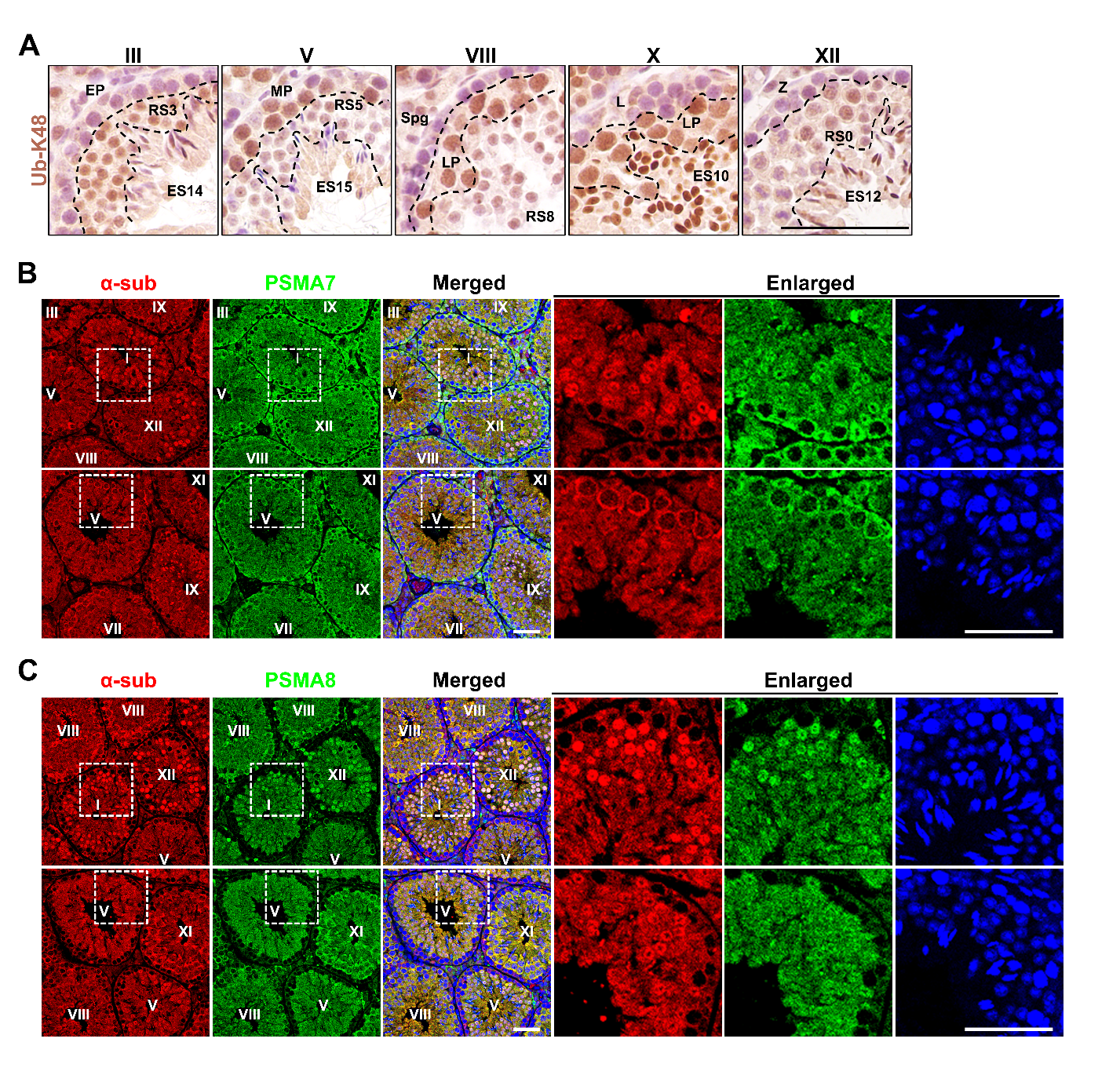
Fig. S1. Patterns of poly-ubiquitination and 20S proteasome α subunits during spermiogenesis.** (**A**) Immunohistochemical (IHC) staining of lysine 48-linked ubiquitin (Ub-K48) during spermatogenesis. Roman numerals indicate the stages of seminiferous tubules. Spg, spermatogonia; L, leptonema; Z, Zygonema; EP, early-pachynema; MP, mid-pachynema; LP, late-pachynema; RS, round spermatids; ES, elongating spermatids; SP, Spermatozoa. Scale bar, 50 μm. (**B-C**) Immunofluorescent staining of the 20S proteasome components, α-subunits (α-sub, red) with PSMA7 (B, green) or PSMA8 (C, green) in sections derived from adult wild-type (WT) testes. The regions bordered with a dashed box are enlarged on the right. Scale bars, 50 μm.

**
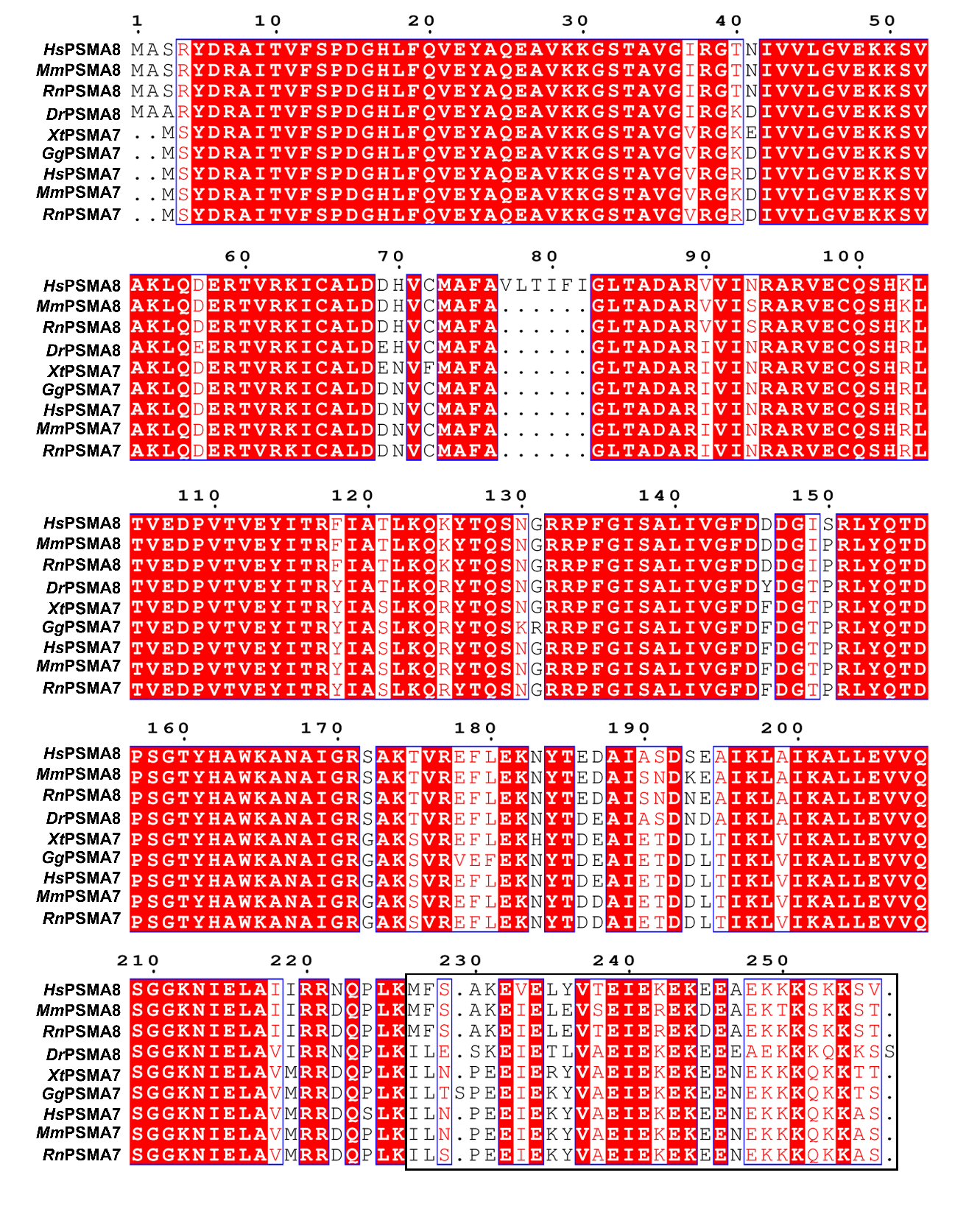
**

**Fig. S2. Alignment of PSMA7 and PSMA8 amino acids sequences among species.** Fully conserved amino acid residues are shown in white on a red background, whereas partially conserved residues are shown in red within blue boxes. The C-terminal 30 amino acids are indicated in a black box. *Hs*, *Homo sapiens*; *Mm*, *Mus musculus*; *Rn*, *Rattus norvegicus*; *Dr*, *Danio rerio*; *Xt*, *Xenopus tropicalis*; *Gg*, *Gallus gallus*.

**
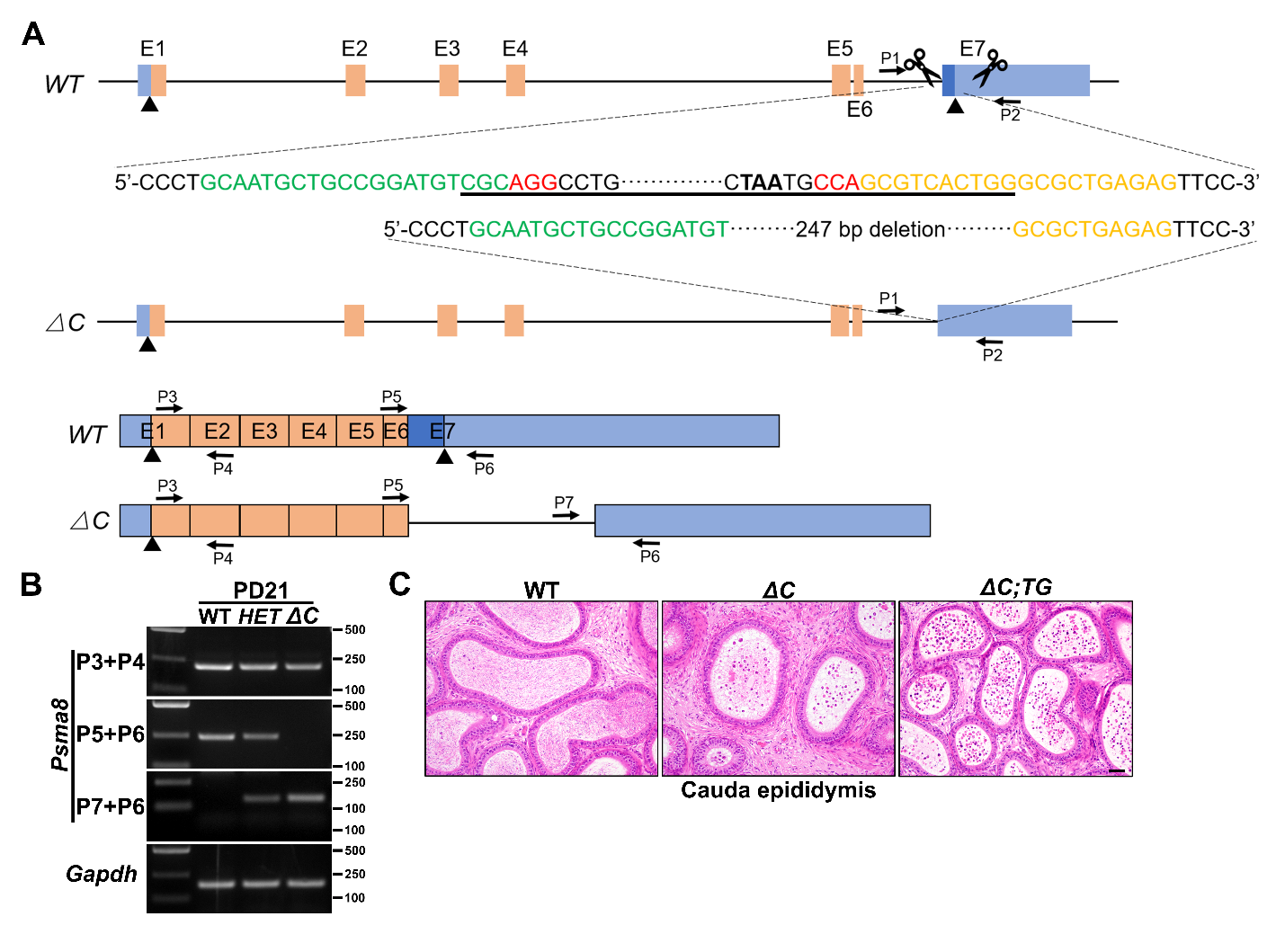
**

**Fig. S3. Generation and validation of *Psma8^ΔC^* mice.** (**A**) Schematic diagram depicting the gene structure of mouse *Psma8*, the strategy to generate *Psma8^ΔC^* allele, as well as the mRNA transcribed by *Psma8^WT^* and *Psma8^ΔC^* alleles. The exons, translation start codon and translation stop codon are indicated. The locations of primers are indicated. (**B**) Semi-quantitative real-time PCR results of mRNAs derived from WT, *HET* and *ΔC* testes at indicated ages. The primers (P3-P7) spanning different regions of the Psma8 WT or *ΔC* mRNAs locations of primers are shown in (A). (**C**) Hematoxylin & eosin (HE) staining of epididymes sections derived from WT, *ΔC* and *ΔC;TG* males at PD42. Scale bar, 50 μm.

**
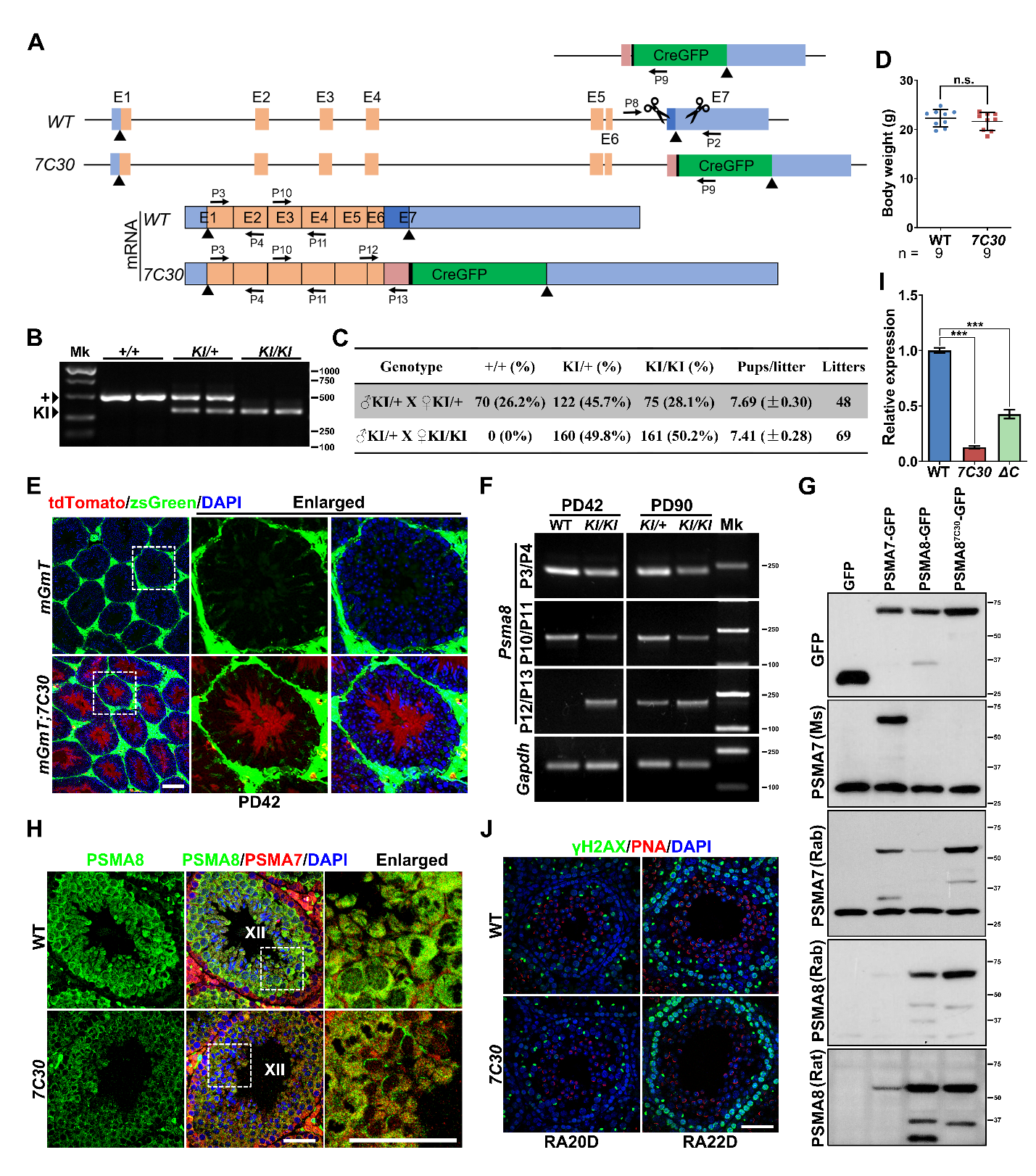
Fig. S4. Generation and validation of the *7C30* knock-in (KI) mice.** (**A**) Schematic diagram showing the strategy to generate *Psma8-7C30* knock-in allele, as well as the mRNA transcribed by WT and *7C30* alleles of *Psma8*. The locations of primers are indicated. (**B**) A representative image showing the genotyping results of the WT (*+/+*), *Psma8^7C30/+^* (*KI/+*) and *Psma8^7C30/7C30^* (*KI/KI*) mice. Mk, DNA marker, and the numbers on the right indicates the size of markers (bp). (**C**) Statistical analysis of litter sizes and ratios of pup genotypes from *KI/+* to *KI/+* breeding and *KI/+* to *KI/KI* breeding. (**D**) Body weights of WT and *7C30* males at PD42. Numbers of mice analyzed (n) are indicated. Error bars indicate S.D. n.s., not significant (two-tailed Student’s *t* test). (**E**) Validation of the CRE activity in the testes derived from the *mGmT;7C30* males at PD42. Scale bar, 100 μm. (**F**) Semi-quantitative real-time PCR results of mRNAs derived from WT, *KI/+* and *KI/KI* testes at indicated ages. The primers (P1-P9) spanning different regions of the *Psma8* WT/ or *7C30* mRNAs locations of primersare shown in Fig. 1A. (**G**) Validation of antibodies against PSMA7 and PSMA8 in detecting exogeneous GFP-tagged PSMA7, PSMA8 and PSMA8-7C30 in HEK293 cells. (**H**) Co-staining of PSMA8 (green) and PSMA7 (red) in WT and *7C30* testes sections at PD42. Scale bar, 50 μm. (**I**) Quantitative real-time PCR results of mRNAs derived from WT, *KI/KI*, and *ΔC* PD42 testes. Error bars indicate S.E.M. ***, P < 0.001 (two-tailed Student’s *t* test). (**J**) Co-staining of γH2AX (green) and PNA (red) in WT and *7C30* testes sections at RA20D and RA22D. Scale bar, 50 μm.

**
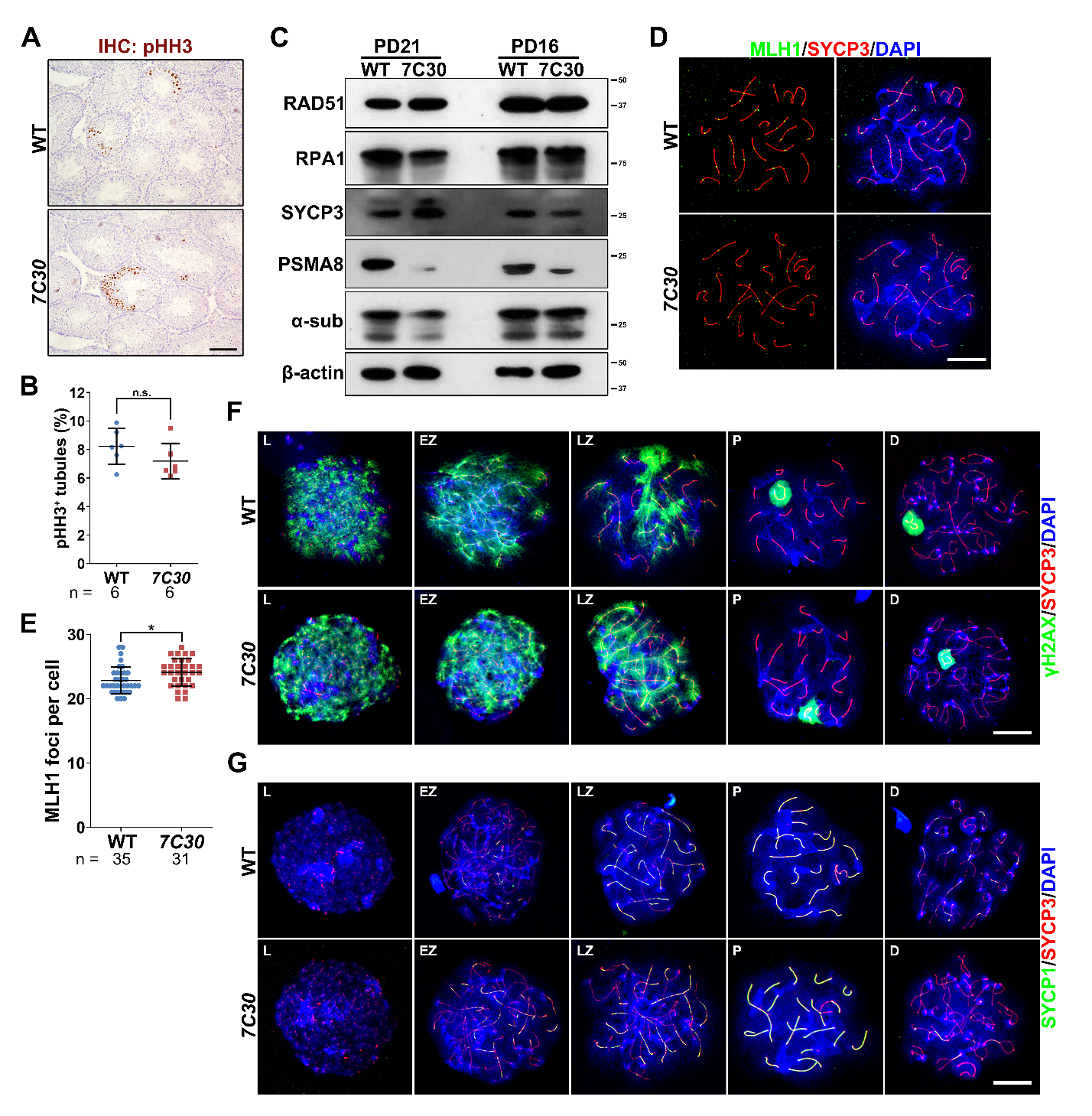
Fig. S5. Meiotic prophase I progression is less affected in *7C30* males.** (**A**) IHC staining of phosphorylated histone H3 (pHH3) in testes sections derived from PD42 WT and *7C30* males. Scale bar, 100 μm. (**B**) Quantification of the percentages of pHH3-positive seminiferous tubules in WT and *7C30* males at PD42. Error bars indicate S.D. n indicates the number of sections analyzed. n.s., not significant (two-tailed Student’s *t* test). (**C**) Western blotting data with antibodies against indicated proteins in testes derived from WT and *7C30* males at PD16 and PD21. (**D-E**) IF staining (D) and the quantification (E) of the MLH1 foci (green) contra-stained with SYCP3 (red) on nuclear surface spreads prepared from WT and *7C30* testes. Scale bar, 10 μm. Error bars indicate S.D. n indicates the numbers of cells analyzed. *, P < 0.05 (two-tailed Student’s *t* test). (**F-G**) Co-staining of γH2AX (F, green) or SYCP1 (G, green) with SYCP3 (red) on the nuclear surface spreads of spermatocytes derived from WT and *7C30* males at PD42. Scale bars, 10 μm.

**
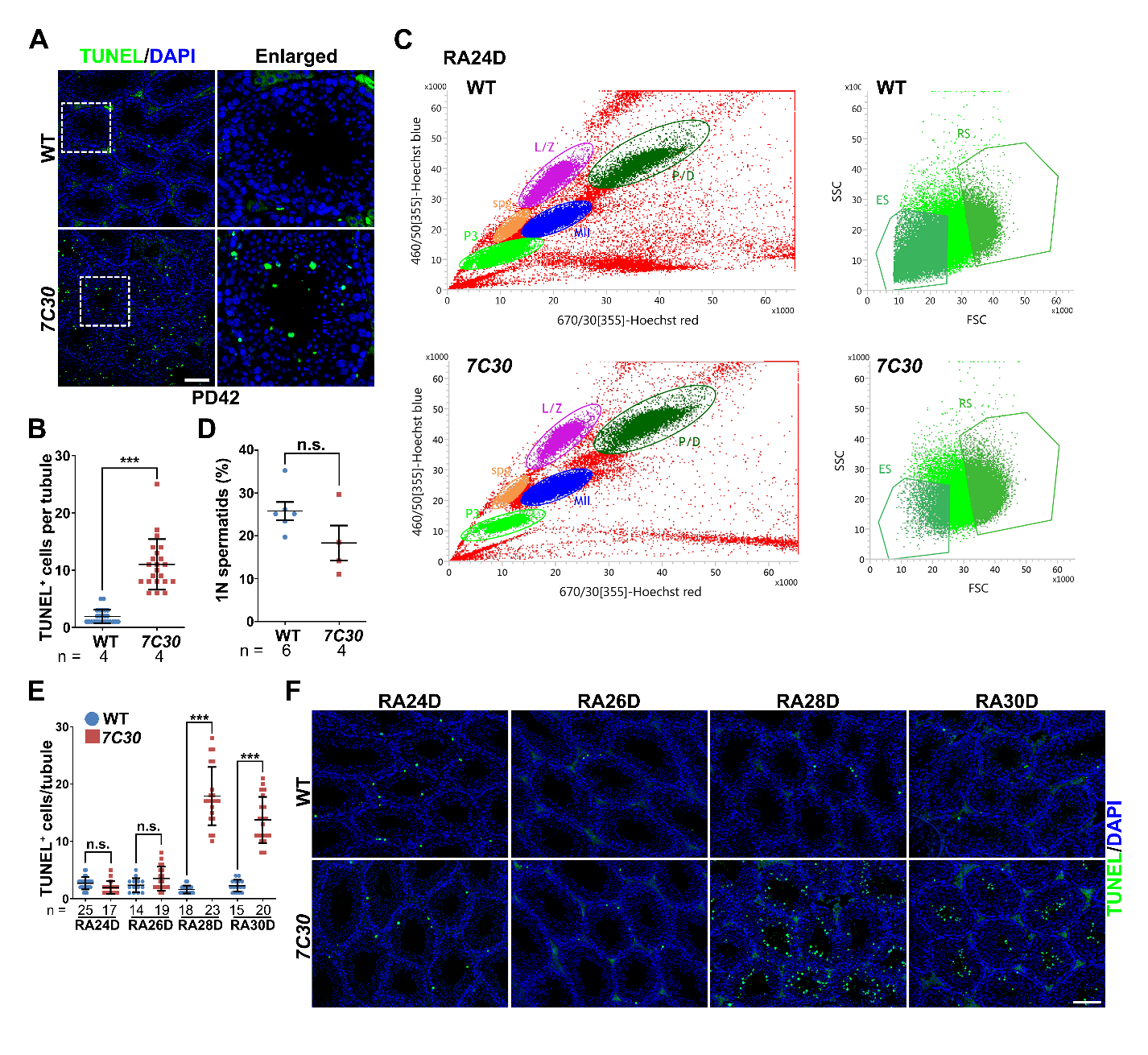
Fig. S6. Synchronized male germ cells exhibits delayed spermiogenesis in *7C30* males.** (**A**) TUNEL assays showing the apoptotic cells in WT and *7C30* testes at PD42. Scale bar, 100 μm. (**B**) Quantification of TUNEL-positive cells per seminiferous tubule. Error bars indicate S.D. n indicates the numbers of sections analyzed. ***, P < 0.001 (two-tailed Student’s *t* test). (**C**) Representative images showing the distribution of male germ cells in FACS, plotted by Hoechst red and Hoechst blue. WT and *7C30* males are treated with WIN18446 and RA for spermatogenesis synchronization and sacrificed at 24 days post RA (RA24D). (**D**) Quantification of the haploid spermatids in WT and *7C30* testes at RA24D. Error bars indicate S.D. n indicates the numbers of mice analyzed. n.s., not significant (two-tailed Student’s *t* test). (**E**) TUNEL assay showing the apoptotic cells in WT and *7C30* testes at RA24D-RA30D. Scale bar, 100 μm. (**F**) Quantification of TUNEL-positive cells per seminiferous tubule. Error bars indicate S.D. n indicates the numbers of sections analyzed. n.s., not significant and ***, P < 0.001 (two-tailed Student’s *t* test).

**
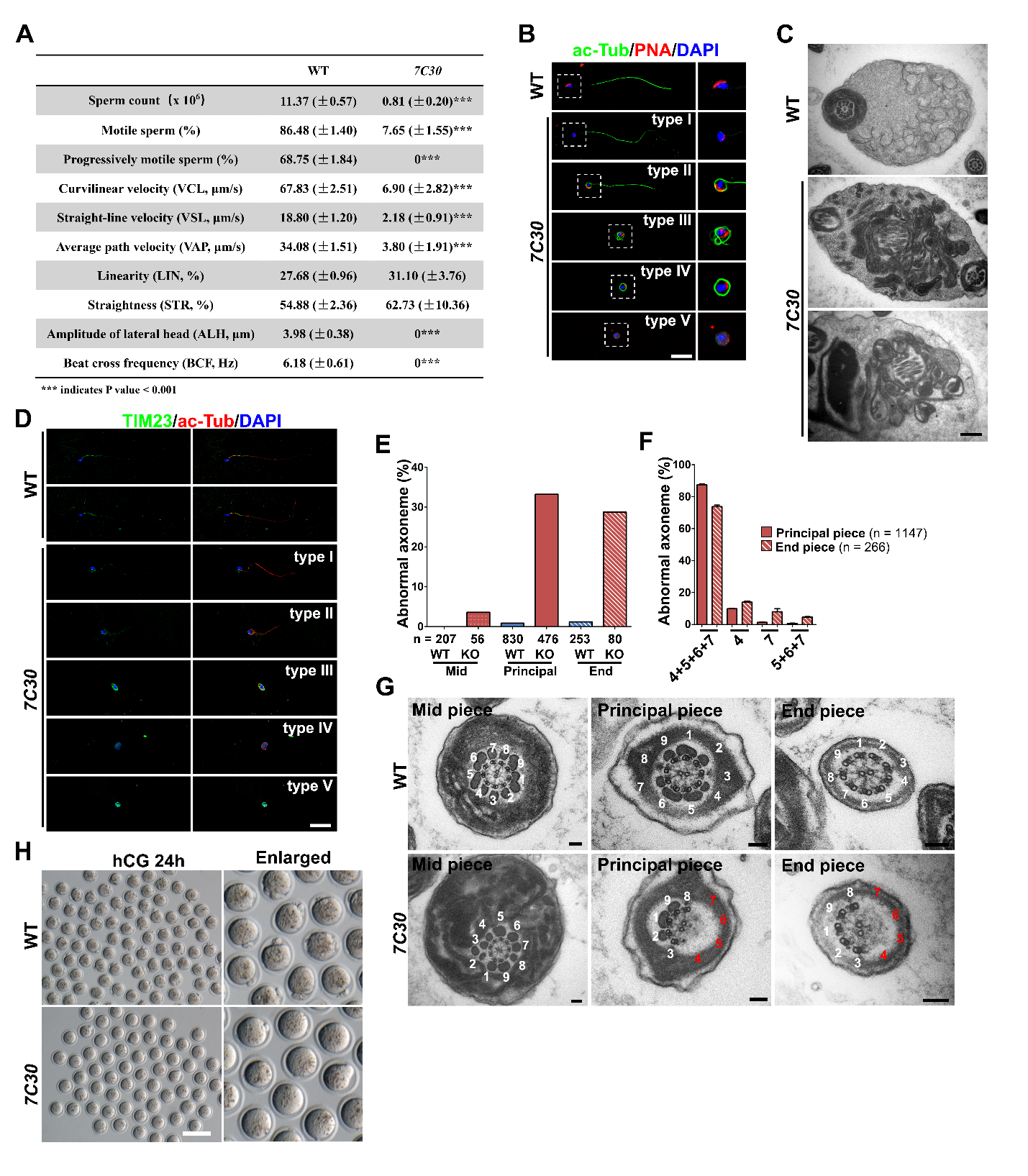
** **Fig. S7. *7C30* spermatozoa exhibit morphological abnormities.** (**A**) CASA results showing the examination in quantity and motility of adult WT and *7C30* spermatozoa. Data are provided as mean ± S.E.M. ***, P < 0.001 (two-tailed Student’s *t*-test). (**B**) IF staining of acetylated-α-tubulin (ac-Tub) and PNA in WT and *7C30* spermatozoa. Scale bar, 20 μm. (**C**) Transmission electric microscopy (TEM) images showing abnormal mitochondrial morphology of *7C30* spermatozoa. Scale bar, 500 nm. (**D**) IF staining of TIM23 (a mitochondrial inner membrane protein, green) and ac-Tub (red) in WT and *7C30* spermatozoa. Scale bar, 20 μm. (**E-F**) Quantification of abnormal axonemes in WT and *7C30* spermatozoa. Error bars indicate S.E.M. n indicates the numbers of spermatozoa analyzed. (**G**) TEM images showing the morphology of mid-piece, principal piece and end piece of WT and *7C30* spermatozoa. Scale bars, 100 nm. (**H**) *In vitro* fertilization (IVF) experiments of spermatozoa derived from WT and *7C30* males with WT oocytes at the MII stage. Scale bar, 100 μm.

**
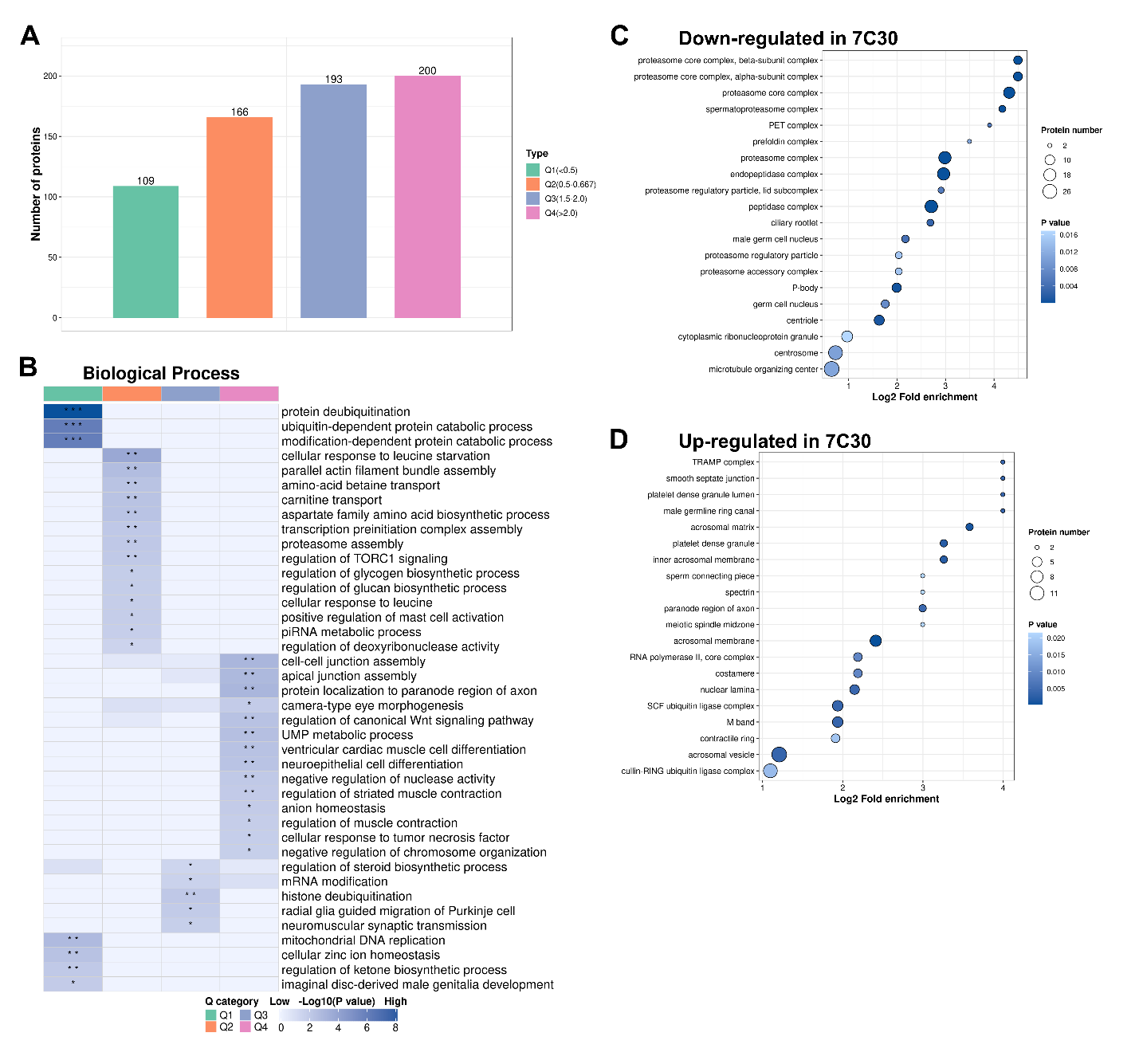
**

**Fig. S8. Gene Ontology enrichment analyses of regulated proteins in *7C30* spermatids.**

**
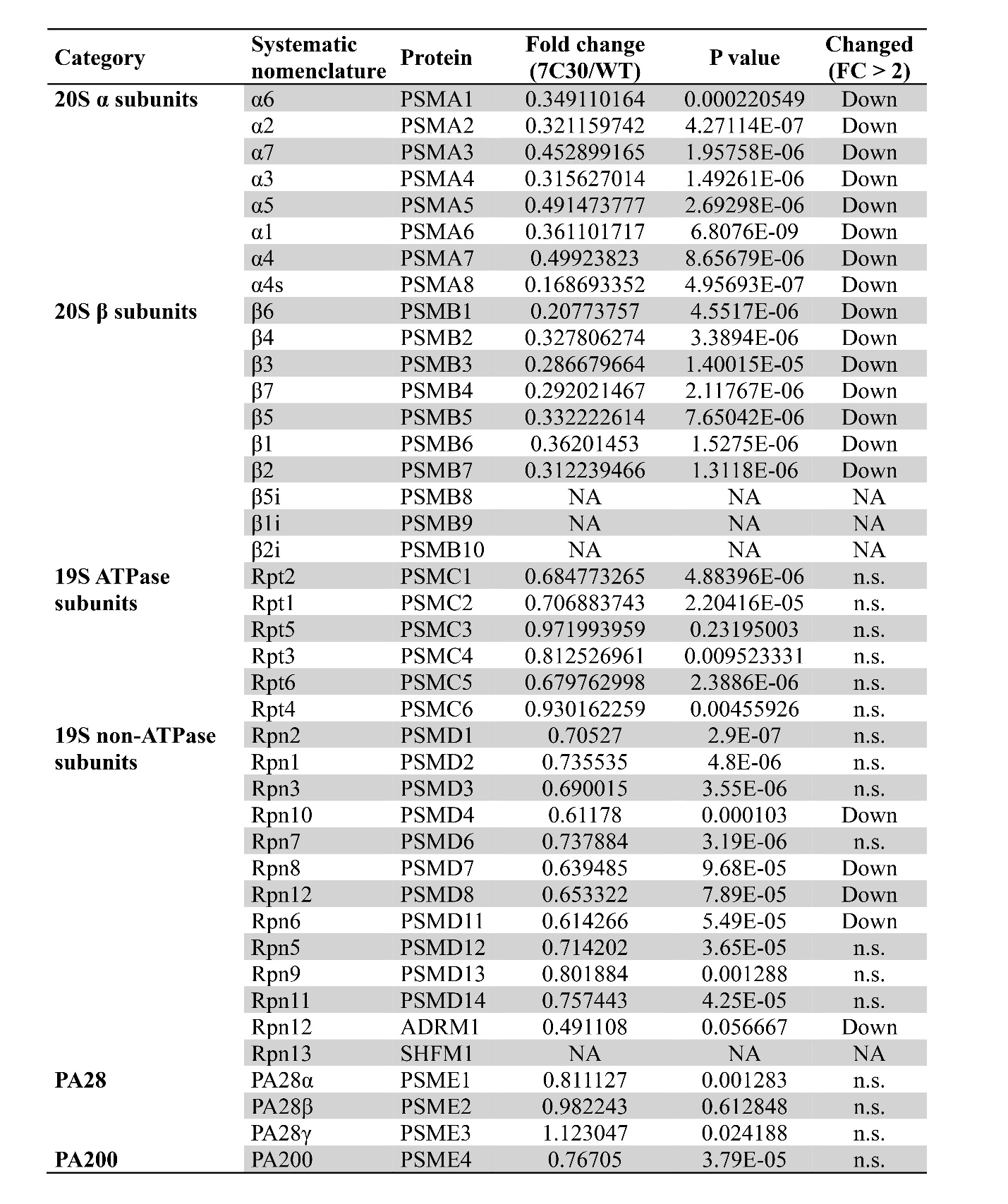
**

**Fig. S9. Fold change of proteasome subunits in *7C30* spermatids at RA24D.**

**
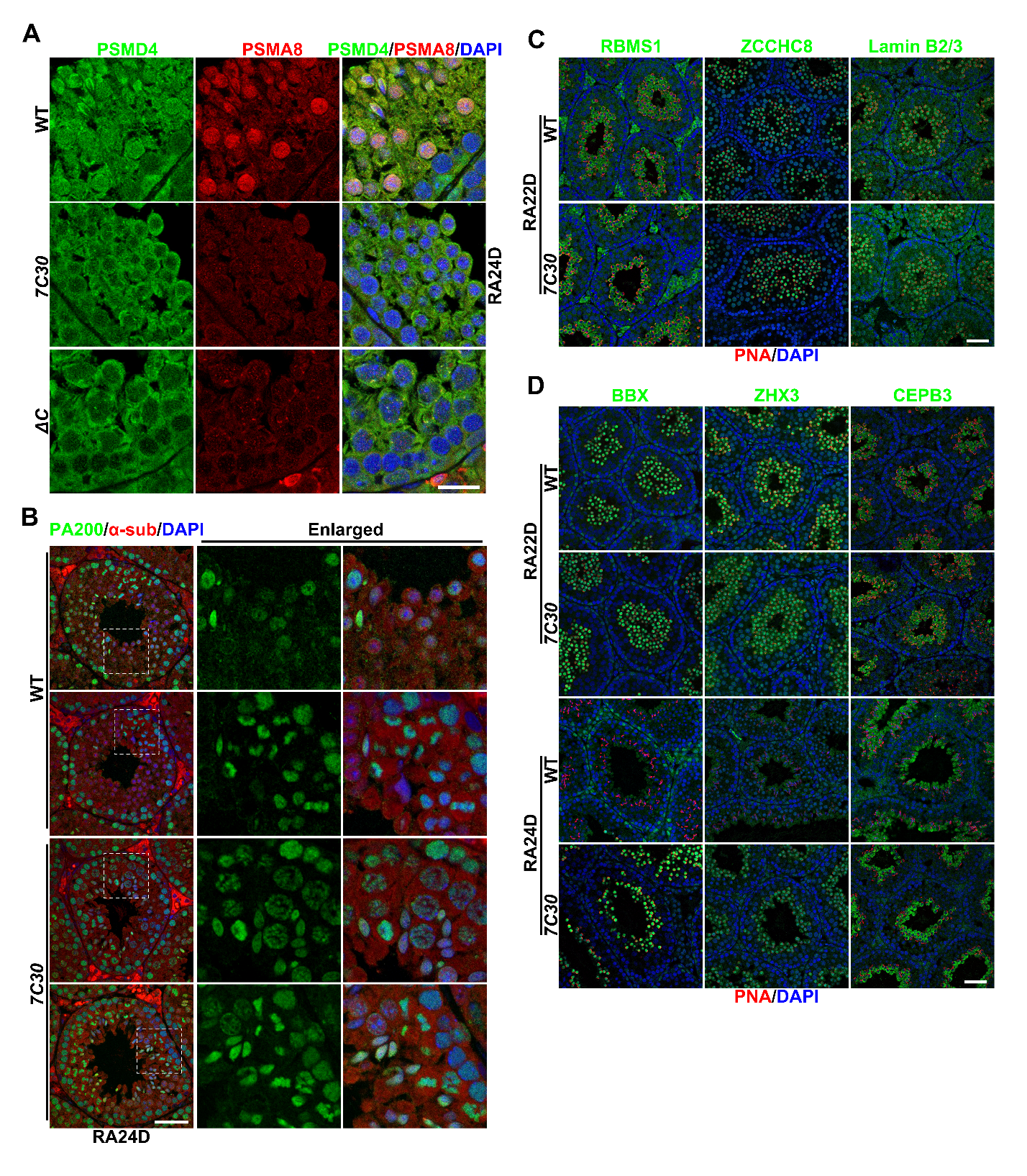
Fig. S10. IF staining of RPs and s20S target proteins in WT and *7C30* testes.** (**A**) Co-staining of PSMD4 (green) and PSMA8 (red) in testes sections derived from synchronized WT, *7C30* and *ΔC* testes at RA24D. Scale bar, 20 μm. (**B**) Co-staining of PA200 (PSME4, green) and α-sub (red) in testes sections derived from synchronized WT and *7C30* testes at RA24D. Scale bar, 50 μm. (**C-D**) IF staining of s20S target proteins showing their stabilization in *7C30* testes. Scale bars, 50 μm.


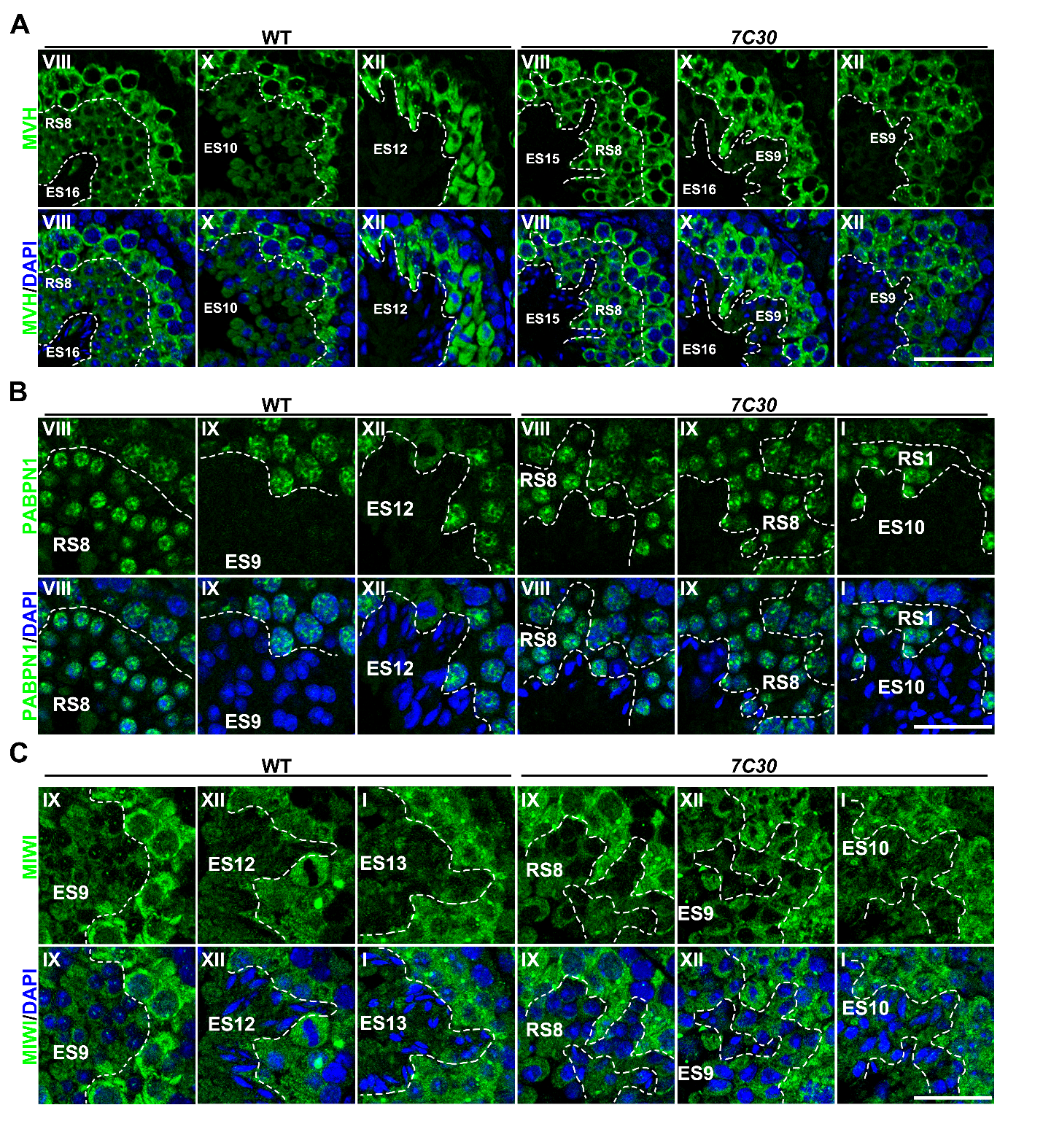
**Fig. S11. IF staining of MVH (A), PABPN1 (B) and MIWI (C) in testes sections derived from WT and 7C30 males at PD42.** Scale bars, 50 μm.

**
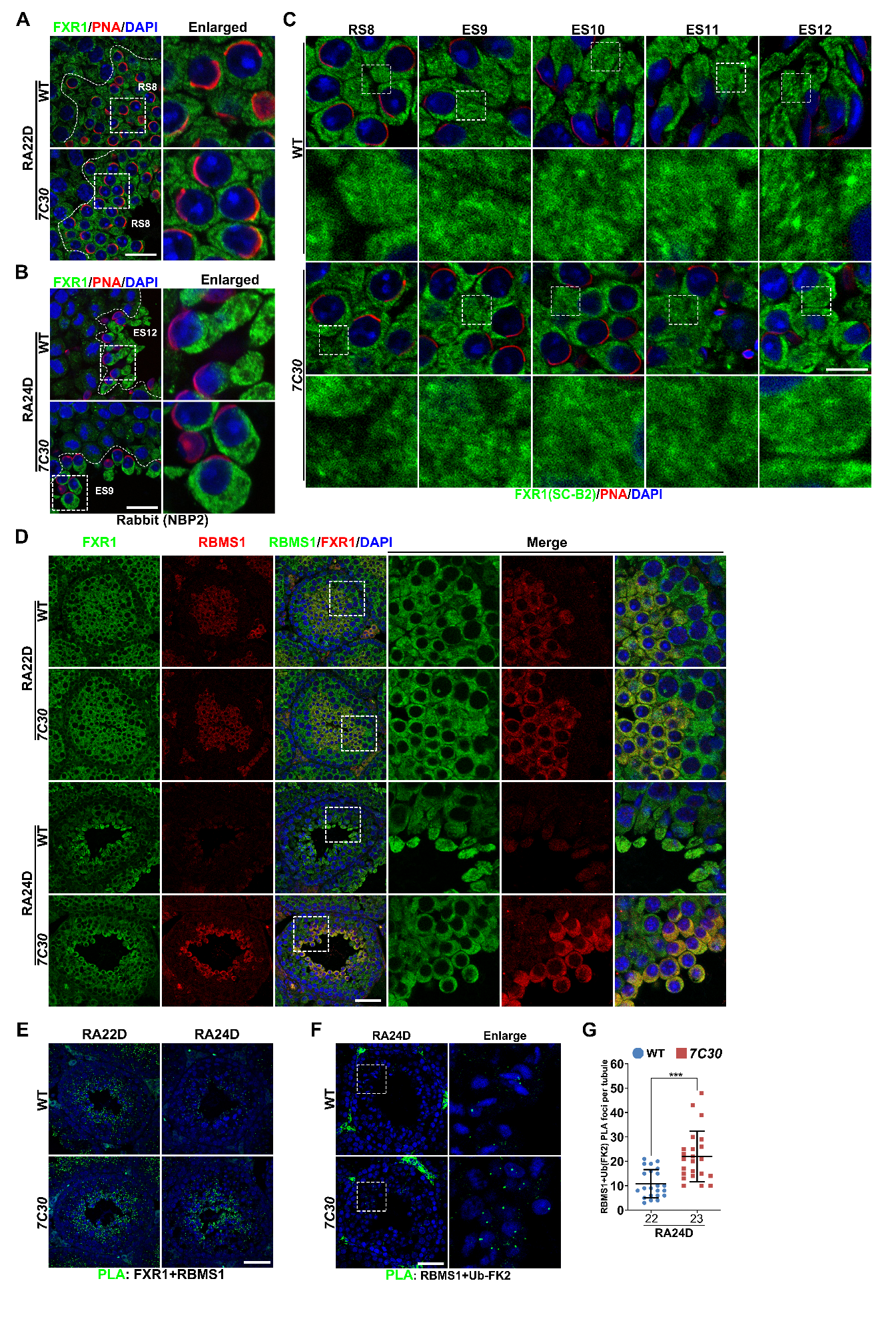
**

**Fig. S12. Impaired LLPS of FXR1 in *7C30* spermatids.** (**A**) IF staining of FXR1 in testes sections derived from WT and *7C30* males at RA22D. Scale bar, 20 μm. (**B**) IF staining with a rabbit anti-FXR1 antibody in testes sections derived from WT and *7C30* males at RA24D. Scale bar, 20 μm. (**C**) IF staining of FXR1 at different ES stages in testes sections derived from WT and *7C30* males at PD42. The framed cytoplasm of spermatids are enlarged. Scale bar, 10 μm. (**D**) Co-staining of FXR1 (green) and RBMS1 (red) in WT and *7C30* spermatids at RA22D and RA24D. Scale bar, 10 μm. (**E**) PLA was carried out with the combination of RBMS1 (rabbit) and FXR1 (mouse) antibodies in WT and *7C30* testes sections at RA22D and RA24D. Scale bar, 50 μm. (**F**) PLA was performed with RBMS1 (rabbit) and Ub-FK2 (mouse) antibodies in WT and *7C30* testes sections at RA24D. Scale bar, 50 μm. (**G**) Quantification of foci per seminiferous tubule. Error bars indicate S.D. n indicates the numbers of sections analyzed. ***, P < 0.001 (two-tailed Student’s *t* test).


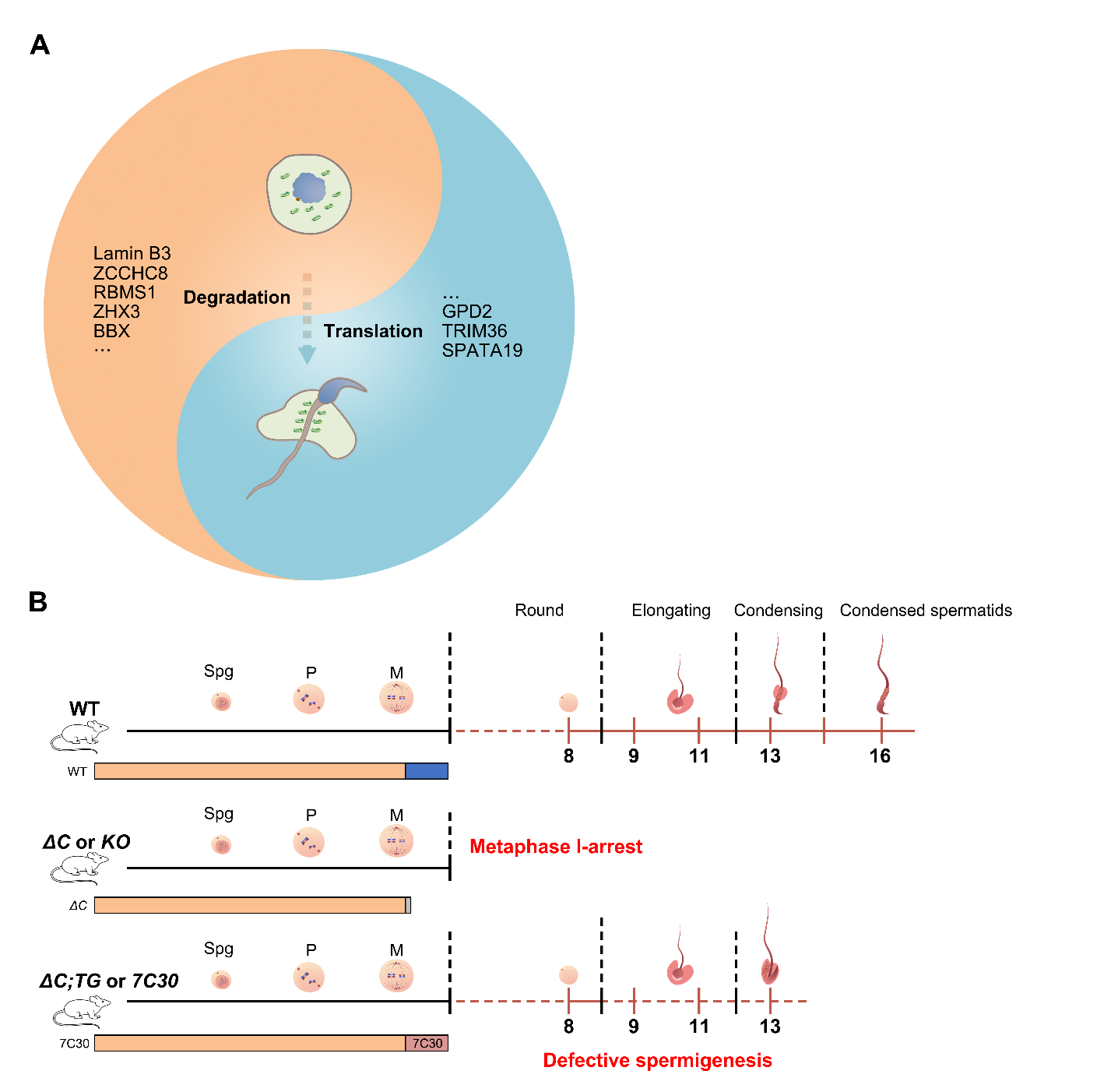


**Fig. S13. Summary of the molecular mechanism and phenotypes of s20S in spermiogenesis in this study.**  (**A**) Schematic diagram elucidating the dynamic changes of proteome through extensive protein degradation and translation during spermiogenesis. (**B**) Schematic diagram summarizing the protein domains and spermatogenic defects in *Psma8^ΔC^*, *Psma8^ΔC;TG^* and *Psma8^7C30^* testes.

**Table S3.** Primer sequences.

| **Primer name** | **Genes targeted** | **Application** | **Sequences (5’-3’)** |
| --- | --- | --- | --- |
| Z124 | *H11-CAG-Psma8* | Genotyping (WT, 412 bp; *TG*, 266 bp) | 5’-CAGCAAAACCTGGCTGTGGATC-3’ |
| Z125 |  |  | 5’-ATGAGCCACCATGTGGGTGTC-3’ |
| Z503 |  |  | 5’-GGGCCATTTACCGTAAGTTATG-3’ |
| P1 | *Psma8-ΔC* | Genotyping (WT, 589 bp; *ΔC*, 316 bp) | 5’-GTCTGTGGGAATCATCTACATGC-3’ |
| P2 |  |  | 5’-GATTAGAGTTACGTGGAACTGAGG-3’ |
| P3 | *Psma8* | RT-PCR (220 bp) | 5’-GGTATGACAGGGCAATCACC-3’ |
| P4 |  |  | 5’-CCTGCAAAAGCCATGCAGAC-3’ |
| P5 | *Psma8* | RT-PCR (248 bp) | 5’-TCTGGCGGAAAGAACATTG-3’ |
| P6 |  |  | 5’-ATTCTTGAAGATGAACGTAG-3’ |
| P6 | *Psma8* | RT-PCR (133 bp) | 5’-ATTCTTGAAGATGAACGTAG-3’ |
| P7 |  |  | 5’-gatgtgtaaggaagtatcat-3’ |
| P8 | *Psma8-7C30* | Genotyping (WT, 482 bp; *7C30*, 301 bp) | 5’-ACATGTCACAGGGTGACAGTGT-3’ |
| P2 |  |  | 5’-GATTAGAGTTACGTGGAACTGAGG-3’ |
| P9 |  |  | 5’-ACAACCACTACCTGAGCACCC-3’ |
| P10 | *Psma8* | RT-PCR (209 bp) | 5’-ATGCTAGAGTGGTGATCAG-3’ |
| P11 |  |  | 5’-ATCTGTCTGATACAATCTTG-3’ |
| P12 | *Psma8* | RT-PCR (210 bp) | 5’-GCCGAAGTGCTAAAACTGTC-3’ |
| P13 |  |  | 5’-TTTCAGCAACATACTTCTCA-3’ |
| Z531 | *Gapdh* | RT-PCR (181 bp) | 5’-ACACTGAGGACCAGGTTGTCTC-3’ |
| Z532 |  |  | 5’-TACTCCTTGGAGGCCATGTAG-3’ |
| Y750 | *Gpd2* | Template generation (2209 bp) | 5’-GTGATCAGGCTGAGCAATGG-3’ |
| Y751 |  |  | 5’-TGGTCAGACTCACAATCCTCC-3’ |
| P14 |  | RT-PCR (254 bp) | 5’-GCATATGTGGAAGCAGCAGG-3’ |
| P15 |  |  | 5’-AGGTATCGCACACCACCGTG-3’ |
| Y746 | *Spata19* | Template generation (465 bp) | 5’-ATGATCATTACAACATGGATTAT-3’ |
| Y747 |  |  | 5’-TTAGCATTCTGAGCAAGAAG-3’ |
| P16 |  | RT-PCR (228 bp) | 5’-GCTTCTCGGAGCATAAGGGAG-3’ |
| P17 |  |  | 5’-TTCCTGCATGACGTCATCCA-3’ |
| Y748 | *Trim36* | Template generation (2202 bp) | 5’-TAAAGACTCCGTGGTTAATGTCAC-3’ |
| Y749 |  |  | 5’-TGTCCAAGCTACACATCCTCTTC-3’ |
| P18 |  | RT-PCR (264 bp) | 5’-CGTGCAAGGAGCTGTTTACC-3’ |
| P19 |  |  | 5’-CCACATCATGTTCACAGCCA-3’ |

**Table S4.** Antibody information.

| **Protein name** | **Manufacture (catalogue number)** | **Origin** | **Applications**  **(working dilution)** | **Website Link** |
| --- | --- | --- | --- | --- |
| α-tubulin | Merck (F2168) | Mouse | IF (1: 200) | https://www.sigmaaldrich.cn/CN/zh/product/sigma/f2168?context=product |
| Acetylated-α-tubulin | Merck (T7451) | Mouse | IF (1: 500) | https://www.sigmaaldrich.cn/CN/zh/product/sigma/t7451?context=product |
| Phalloidin | Merck (P1951) | Mouse | IF (1: 200) | https://www.sigmaaldrich.cn/CN/zh/product/sigma/p1951?context=product |
| Ub-K48 | Abcam (ab140601) | Rabbit | IHC (1:200) | https://www.abcam.cn/products/primary-antibodies/ubiquitin-linkage-specific-k48-antibody-ep8589-ab140601.html |
| Ubiquitin-FK2 | UBIQUIGENT(#30124) | Mouse | PLA (1:50) |  |
| Ub Ms | CST (3936S) | Mouse | WB (1:500) | https://www.cellsignal.cn/products/primary-antibodies/ubiquitin-p4d1-mouse-mab/3936 |
| PSMA8 | Proteintech (14022-1-AP) | Rabbit | IF (1: 200)  WB (1:1000)  PLA (1: 200) | https://www.ptglab.com/Products/PSMA8-Antibody-14022-1-AP.htm |
| PSMA7 Rab | Proteintech (15219-1-AP) | Rabbit | IF (1:200)  WB (1:1000) | https://www.ptgcn.com/products/OGDH-Antibody-15212-1-AP.htm |
| PSMA7 Ms | Abcam (ab55626) | Mouse | IF (1:100)  WB (1:500)  PLA (1: 100) |  |
| α-sub | Calbiochem  (ST1049-100ul) | Mouse | IF (1:100)  WB (1:500) PLA (1:100) | https://www.sigmaaldrich.cn/CN/zh/product/mm/st1049 |
| PA200 | Abcam (ab181203) | Rabbit | WB (1:200)  IF (1:1000) | https://www.abcam.cn/products/primary-antibodies/proteasome-activator-subunit-4psme4-antibody-epr13577b-c-terminal-ab181203.html |
| PSMC2 | Proteintech (14905-1-AP) | Rabbit | IF (1:200)  WB (1:1000)  PLA (1:200) | https://www.ptgcn.com/Products/PSMC2-Antibody-14905-1-AP.htm |
| PSMD4 | Proteintech (66179-1-Ig) | Mouse | PLA (1:200) | https://www.ptgcn.com/products/PSMD4-Antibody-66179-1-Ig.htm |
| β-actin | Shanghai Genomics (GNI4110-BA) | Mouse | WB (1:1000) | http://www.gnimission.com/resource/article/206 |
| γH2AX | Cell Signaling  (9718S) | Rabbit | IF (1: 400) | https://www.cellsignal.com/products/primary-antibodies/phospho-histone-h2a-x-ser139-20e3-rabbit-mab/9718?N=4294956287&Ntt=h2a.x&fromPage=plp |
| PNA-rhodamine | Molecular Probes (L32458) | NA | IF (1:200) | https://www.thermofisher.cn/order/catalog/product/cn/zh/L32458 |
| RBMS1 | Proteintech (11061-2-AP) | Rabbit | IF (1:200)  WB (1:1000)  PLA (1:50) | https://www.ptgcn.com/products/RBMS1-Antibody-11061-2-AP.htm |
| BBX | Proteintech (17254-1-AP) | Rabbit | IF (1:200)  WB (1:1000) | https://www.ptgcn.com/products/BBX-Antibody-17254-1-AP.htm |
| ZHX3 | Proteintech (29397-1-AP) | Rabbit | IF (1:200)  WB (1:1000) | https://www.ptgcn.com/products/ZHX3-Antibody-29397-1-AP.htm |
| ZCCHC8 | Proteintech (23374-1-AP) | Rabbit | IF (1:200)  WB (1:1000) | https://www.ptgcn.com/products/ZCCHC8-Antibody-23374-1-AP.htm |
| RBM7 | Proteintech (21896-1-AP) | Rabbit | WB (1:1000) | https://www.ptgcn.com/products/RBM7-Antibody-21896-1-AP.htm |
| Lamin B2/3 | Proteintech (10895-1-AP) | Rabbit | IF (1:200)  WB (1:1000) | https://www.ptgcn.com/products/LMNB2-Antibody-10895-1-AP.htm |
| SPATA19 | Proteintech (16656-1-AP) | Rabbit | IF (1:200)  WB (1:1000) | https://www.ptgcn.com/products/SPATA19-Antibody-16656-1-AP.htm |
| TRIM36 | Proteintech (25913-1-AP) | Rabbit | IF (1:200)  WB (1:1000) | https://www.ptgcn.com/products/TRIM36-Antibody-25913-1-AP.htm |
| TIM50 | Abcam (ab109527) | Rabbit | IF (1:200) | https://www.abcam.com/products/primary-antibodies/tim50-antibody-epr5784-ab109527.html |
| GFP | Shanghai Genomics (GNI4110-GP) | Mouse | WB (1:1000) | http://www.gnimission.com/resource/article/88 |
| MLH1 | BD (551092) | Mouse | IF (1: 200) | http://www.bdbiosciences.com/us/applications/research/apoptosis/purified-antibodies/purified-mouse-anti-mlh-1-with-control/p/551092 |
| SYCP3 | homemade | Rat | IF (1:500) | Immunogen: Full length of mouse SYCP3 |
| pHH3 | CST (9701S) | Rabbit | IHC (1:200) | https://www.cellsignal.com/products/primary-antibodies/phospho-histone-h3-ser10-antibody/9701 |
| SYCP1 | Abcam (ab15087) | Rabbit | IF (1:200) | https://www.citeab.com/antibodies/771942-ab15087-anti-scp1-antibody |
| RAD51 | Abcam (ab176458) | Rabbit | IF (1: 200) | http://www.abcam.com/rad51-antibody-chip-grade-ab176458.html |
| RPA1 | Abcam (ab87272) | Rabbit | WB (1:1000) | https://www.abcam.cn/products/primary-antibodies/rpa70-antibody-ab87272.html |
| FLAG | Sigma-aldrich  (F3165) | Mouse | WB (1: 3000) | http://www.sigmaaldrich.com/catalog/product/sigma/f3165?lang=en&region=SE |
| CPEB3 | Proteintech (12669-1-AP) | Rabbit | IF (1:200)  WB (1:1000) | https://www.ptgcn.com/products/CPEB3-Antibody-12669-1-AP.htm |
| PABPN1 | Bethyl (A303-523A-M) | Rabbit | IF (1:200) | https://www.thermofisher.cn/cn/zh/antibody/product/PABPN1-Antibody-Polyclonal/A303-523A |
| MIWI | Proteintech (15659-1-AP) | Rabbit | IF (1:200) | https://www.ptgcn.com/products/PIWIL1-Antibody-15659-1-AP.htm |
| GAPDH | GNI (GNI4110-GH) | Mouse | WB (1:10000) | http://www.gnimission.com/resource/article/76 |
| MVH | Abcam (ab13840) | Rabbit | IF (1: 200);  WB (1: 1000) | http://www.abcam.com/ddx4--mvh-antibody-ab13840.html |
| WT1 | Abcam (ab89901) | Rabbit | IF (1: 200) | https://www.abcam.com/wilms-tumor-protein-antibody-can-r9ihc-56-2-ab89901.html |
| PLZF | Santa Cruz (sc28319) | Mouse | IF (1: 200) | https://www.scbt.com/p/plzf-antibody-d-9/ |
| FXR1 Rab | Novus (NBP2-22246) | Rabbit | WB (1: 1000) | https://www.novusbio.com/products/fxr1-antibody_nbp2-22246 |
| FXR1 Ms | Santa Cruz (sc-374148) | Mouse | IF (1: 100) | https://www.scbt.com/zh/p/fxr1-antibody-b-2 |
| GPD2 | Proteintech (17219-1-AP) | Rabbit | IF (1:200);  WB (1: 1000) | https://www.ptgcn.com/products/GPD2-Antibody-17219-1-AP.htm |
